# Coenzyme Q10 as an adjunctive strategy to reduce paclitaxel-induced toxicities in breast cancer: a randomized controlled trial

**DOI:** 10.1186/s40360-026-01163-7

**Published:** 2026-07-04

**Authors:** Gehad Hassoub, Noha A. El‑Bassiouny, Yasser Abdelkader, Ahmed Ashour Badawy, Amira B. Kassem

**Affiliations:** 1 Department of Clinical Pharmacy and Pharmacy Practice, Faculty of Pharmacy, Damanhour University, Damanhour, Egypt; 2 Oncology Department, Damanhour Oncology Center, Specialized Medical Center, Ministry of Health, Damanhour, Egypt; 3 Department of Clinical Oncology, Faculty of Medicine, Alexandria University, Alexandria, Egypt

**Keywords:** Coenzyme Q10, Paclitaxel, Breast cancer, Chemotherapy toxicities

## Abstract

**Background:**

Paclitaxel is an effective chemotherapeutic agent for breast cancer, but its use is often limited by cumulative toxicities linked to mitochondrial dysfunction and oxidative stress. This study investigated whether Coenzyme Q10 (CoQ10) could mitigate paclitaxel-induced adverse events and improve treatment tolerability.

**Patients and methods:**

In this open label randomized controlled trial, 60 patients with breast cancer were randomized (1:1) receive weekly paclitaxel (80 mg/m²) for 12 weeks either alone (control group, n = 30) or in combination with oral CoQ10. The primary outcome was the cumulative incidence of grade ≥ 2 peripheral neuropathy. Secondary endpoints included time-to-onset of grade ≥ 2 neuropathy; fatigue, headache, insomnia, musculoskeletal, gastrointestinal, and hematological adverse events; and left ventricular ejection fraction. Adverse events were graded using the Common Terminology Criteria for Adverse Events (CTCAE), version 5.0.

**Results:**

CoQ10 supplementation was associated with a lower incidence of clinically relevant neuropathy, with grade ≥ 2 events occurring in 68% of the CoQ10 group versus 96% of controls (p = 0.01) with delayed onset of neuropathy (30.0 vs. 20.0 days; log-rank p = 0.005). Significant reductions in severity were also observed for fatigue and insomnia from week 9, and for mucositis, diarrhea, arthralgia, and myalgia from week 11 (p < 0.05). Hemoglobin levels were higher at week 12 (p = 0.009). CoQ10 was associated with preservation of left ventricular ejection fraction (p = 0.005).

**Conclusion:**

CoQ10 supplementation during paclitaxel therapy was associated with reduced treatment-related toxicities, preservation of hematologic parameters, and favorable changes in left ventricular ejection fraction, with favorable tolerability.

**Trial registration:**

ClinicalTrials.gov (NCT06570811) on August 26, 2024. Available at: https://clinicaltrials.gov/study/NCT06570811.

**Supplementary Information:**

The online version contains supplementary material available at 10.1186/s40360-026-01163-7.

## Introduction

According to GLOBOCAN 2022, published by the International Agency for Research on Cancer (IARC) of the World Health Organization (WHO), breast cancer is the second most common malignancy and the second leading cause of cancer-related mortality worldwide [1]. In Egypt, it is the most prevalent cancer among women and the second leading cause of cancer mortality after liver cancer [2, 3]. The anthracycline-taxane combination is one of the most widely used chemotherapy regimen for early and locally advanced breast cancer [4, 5]. However, up to 87% of patients experience chemotherapy-related adverse events during or after treatment [6, 7]. Paclitaxel, a key component of this regimen, is associated with a broad spectrum of toxicities. One of the most clinically important toxicities is peripheral neuropathy, with incidence rates ranging from 11 to 87%, followed by fatigue (11-71.5%) and musculoskeletal pain (up to 58%), all of which markedly impair quality of life [8–12]. Other common adverse events include gastrointestinal toxicities such as nausea, vomiting, and diarrhea; hematologic toxicities such as anemia and neutropenia; and dermatologic toxicities, including alopecia and nail changes [13–20].

Current management of paclitaxel-induced toxicities remains largely supportive [21]. Multiple agents, including gabapentin, metformin, duloxetine, and antioxidants, have been evaluated for preventing paclitaxel-induced peripheral neuropathy (PIPN); however, none have been recommended by the American Society of Clinical Oncology [22–28]. Chemotherapy-related fatigue is mainly managed through non-pharmacological interventions such as exercise and cognitive behavioral therapy, with limited evidence specifically addressing paclitaxel-induced fatigue [29–31]. Glutamine supplementation has been evaluated for the management of paclitaxel-induced arthralgia and myalgia in breast cancer patients; however, results remain conflicting, and treatment continues to depend on symptomatic relief through nonsteroidal anti-inflammatory drugs (NSAIDs) or corticosteroids [12, 32–34]. A comparable pattern is observed with mucositis, for which supportive care, particularly meticulous oral hygiene, remains the main evidence-based approach [17, 35]. The clinical benefits of antioxidants, including vitamin E, zinc, propolis, curcumin, and silymarin, remain inconclusive [36–40]. Dermatological toxicities, including alopecia and nail changes such as ridging and discoloration, are common during taxane therapy [41, 42]. Scalp cooling remains the most established strategy for preventing chemotherapy-induced alopecia, whereas no standardized measures exist for nail toxicity [43]. Despite extensive research, no pharmacological agent has been firmly established for the prevention of multiple chemotherapy-induced toxicities. Serageldin et al. reported that metformin was associated with reductions in neuropathy, mucositis, fatigue, and cardiotoxicity during AC-T therapy (doxorubicin, cyclophosphamide, and paclitaxel) [44].These limitations highlight the ongoing need for safe, broad-spectrum supportive agents capable of mitigating multiple chemotherapy-induced toxicities.

Coenzyme Q10 (CoQ10) has emerged as a promising mitochondrial antioxidant, with increasing preclinical and clinical evidence suggesting a potential role in reducing chemotherapy-induced toxicities. CoQ10 is vital for mitochondrial energy production and serves as a key defense against oxidative stress, particularly in high-energy organs such as the heart, liver, skeletal muscles, and kidney [45]. Clinical evidence suggests that CoQ10 supplementation mitigates anthracycline-related cardiotoxicity, thereby permitting the administration of higher, more efficacious chemotherapy doses [46–48]. CoQ10 supplementation has also been associated with improved antioxidant capacity and reduced oxidative stress in patients after hepatocellular carcinoma treatment [49]. Preclinical studies in rat models also support CoQ10’s protective effects, showing it can mitigate doxorubicin- and gentamicin-induced nephrotoxicity and paclitaxel-induced peripheral neuropathy [50–53]. Given these promising findings this study aimed to investigate whether CoQ10 supplementation can mitigate paclitaxel-related toxicities in patients with breast cancer.

## Patients and methods

### Study design and setting

This parallel-group, open-label randomized controlled trial was conducted at Damanhour Oncology Center. Ethical approval was obtained from the Research Ethics Committee, Faculty of Pharmacy, Damanhour University (IRB No. 823PP66). The trial was registered at ClinicalTrials.gov on August 26, 2024 (identifier: NCT06570811). The study protocol, including predefined primary and secondary endpoints and the statistical analysis plan, were finalized before enrollment of the first participant. Recruitment began in October 2024. No participants were enrolled before registration, and no post-enrollment modifications to outcomes or analysis methods were made. The study protocol and statistical analysis plan are available from the corresponding author upon reasonable request. All procedures were conducted in accordance with the Declaration of Helsinki. Patients and the public were not involved in any aspect of the design, conduct, or reporting of this trial.

### Patients

All participants provided written informed consent before enrollment and were screened for eligibility. Eligible patients were female patients aged 18 years or older with newly diagnosed breast cancer and an Eastern Cooperative Oncology Group (ECOG) performance status of 0 to 2. Exclusion criteria included pregnancy, breastfeeding, hereditary muscle disorders, diabetes mellitus, alcoholism, thyroid dysfunction, metastatic disease, or known allergies to Coenzyme Q10 or related compounds. Patients with advanced liver disease, defined as liver enzymes > 3 times the upper limit of normal or cirrhosis or chronic kidney disease, known allergy to CoQ10 or related compounds were also excluded.

### Randomization and study interventions

All patients first completed four cycles of standard doxorubicin plus cyclophosphamide (AC) protocol, consisting of doxorubicin (60 mg/m²) and cyclophosphamide (600 mg/m²). Patients who remained eligible were then randomly assigned in a 1:1 ratio to either the control group (*n* = 30), which received paclitaxel at 80 mg/m² weekly for 12 weeks, corresponding to four 3-week cycles, or the CoQ10 group (*n* = 30), which received the same paclitaxel regimen combined with oral CoQ10 [49, 54, 55]. The CoQ10 formulation used in this study was Dozova CoQ10 Ubiquinol^®^ (Zeta Pharma, Egypt), supplied as hard gelatin capsules containing 200 mg ubiquinol per capsule. Participants in the CoQ10 group received 200 mg twice daily (total daily dose 400 mg), administered orally with breakfast and dinner to optimize absorption. To minimize selection bias, simple randomization was performed using a computer-generated random sequence. Allocation was concealed using sequentially numbered, opaque, sealed envelopes. These envelopes were opened by a study investigator only after a participant had completed the baseline assessment and provided written informed consent, ensuring that the next treatment assignment could not be predicted in advance.

### Intervention delivery and adherence

Paclitaxel was administered by oncology nurses at the Damanhour Oncology Center. For the intervention group, oral CoQ10 was dispensed to the participants by the clinical pharmacy investigator (GH) during the weekly chemotherapy visits. During the same visits, the physician (YAA or AAB) provided the participants with detailed instructions on the timing and administration of the supplement. Adherence to CoQ10 supplementation was assessed at each chemotherapy cycle using pill counts from returned bottles and review of the participant’s daily dosing diary. Participants were considered adherent if they had taken ≥ 90% of the prescribed doses across the entire 12-week paclitaxel treatment period.

### Study outcomes and monitoring

#### Primary outcome

The primary endpoint was the cumulative incidence of grade ≥ 2 paclitaxel-induced peripheral neuropathy at week 12, assessed according to the National Cancer Institute Common Terminology Criteria for Adverse Events (NCI-CTCAE), version 5.0. This endpoint was selected to quantify the overall risk of clinically significant neuropathy during the treatment period. Weekly assessments of neuropathy severity of any grade were also conducted to evaluate symptom progression over time.

#### Secondary outcomes

Time-to-onset of grade ≥ 2 neuropathy was prespecified as a secondary, supportive time-to-event endpoint to provide additional information on the temporal pattern of neuropathy development. Additional secondary outcomes included non-hematological toxicities, including fatigue, headache, insomnia, arthralgia, myalgia, alopecia, nail changes, and gastrointestinal events such as nausea, vomiting, diarrhea, constipation, and oral mucositis. Hematological parameters included measurement of hemoglobin levels, absolute neutrophil count (ANC), and platelet count. Cardiac function was evaluated by measuring left ventricular ejection fraction via transthoracic echocardiography. Pathologic complete response (pCR) was not a predefined endpoint, as the study primarily focused on treatment-related toxicities rather than oncologic efficacy outcomes.

#### Monitoring and grading procedures

All adverse events were recorded using a standardized assessment form (Supplementary File 1) and graded from 1 to 5 according to the National Cancer Institute Common Terminology Criteria for Adverse Events (NCI-CTCAE), version 5.0. All adverse events, including peripheral neuropathy, were evaluated weekly throughout the 12-week paclitaxel treatment period. Long-term follow-up after treatment completion was not performed; therefore, assessments were restricted to the active treatment duration.

To enhance data capture and reduce recall bias, a dual-modality approach was utilized: structured face-to-face interviews were conducted by a trained physician experienced in oncology clinical trials before each chemotherapy session and reviewed by the study investigator. while structured telephone follow-ups were performed three days after each session to document newly emerging or transient symptoms. In addition, Hematological events were evaluated before each 3-week treatment cycle on laboratory findings and body temperature monitoring. Alopecia and nail changes were assessed at baseline and at week 12. left ventricular ejection fraction was assessed at baseline and after completion of the 12-week treatment period.

### Monitoring of CoQ10-related adverse events

CoQ10-related adverse events were actively monitored throughout the study. During scheduled physician visits and follow-up phone calls, participants in the CoQ10 group were systematically asked about symptoms suggestive of intolerance, including gastrointestinal discomfort, nausea, vomiting, diarrhea, dizziness, photophobia, irritability, headache, insomnia, or any other newly developed symptoms [55].

### Sample size calculation

Sample size estimation was based on the primary endpoint, defined as the cumulative incidence of grade ≥ 2 paclitaxel-induced peripheral neuropathy (PIPN), assessed according to the National Cancer Institute Common Terminology Criteria for Adverse Events (NCI-CTCAE) version 5.0. Based on previously published studies [23, 27], the incidence of grade ≥ 2 neuropathy among patients receiving paclitaxel was estimated to range from 50% to 70%. For the present calculation, a control-group incidence of 60% was assumed. The calculation assumed that CoQ10 supplementation would reduce the incidence of grade ≥ 2 neuropathy to 20%, corresponding to an absolute risk reduction of 40%, which was considered clinically meaningful. The sample size calculation was performed using PASS 2000 (NCSS, LLC, Kaysville, Utah, USA), based on a two-sided chi-square test for comparison of proportions between two independent groups without continuity correction. Assuming a significance level (α) of 0.05 and a statistical power of 80%, a minimum sample size of 50 patients (25 patients per group) was required to detect the anticipated difference. To account for an anticipated dropout rate of 20%, the total sample size was increased to 60 patients. No interim analyses or stopping rules were applied.

### Statistical analysis

Analyses were performed using both a modified intention-to-treat (mITT) and per-protocol (PP) approaches. The mITT population included all patients who initiated paclitaxel treatment and had at least one post-baseline assessment. This population was used for time-to-event analyses, with patients censored at the time of last available follow-up. The PP population included patients who completed the 12-week treatment period (*n* = 51), had adequate treatment exposure, and had no major protocol deviations. This population was used for the primary and other secondary analyses. Missing data for patients who withdrew or were lost to follow-up (*n* = 9) were not imputed. Statistical analyses were conducted using IBM SPSS Statistics (IBM Corp., Armonk, NY, USA). Categorical variables are presented as frequencies and percentages, and continuous variables as mean ± standard deviation (SD) or median [interquartile range (IQR)], as appropriate. Normality was assessed using the Shapiro-Wilk test. Between-group comparisons were performed using the independent samples t-test or Mann-Whitney U test, and within-group comparisons were performed using the paired t-test or Wilcoxon signed-rank test. The primary endpoint, cumulative incidence of grade ≥ 2 peripheral neuropathy, was compared between groups using the Chi-square test, with odds ratios (ORs) and 95% confidence intervals (CIs) reported. Longitudinal neuropathy severity was analyzed using ordinal logistic regression. Secondary endpoints, including time-to-onset of grade ≥ 2 neuropathy were analyzed using Kaplan-Meier survival analysis, with time defined from initiation of paclitaxel to first event. Patients without events were censored at their last assessment, and those lost to follow-up were censored at their last evaluation. Group differences were assessed using the log-rank test, and hazard ratios (HRs) with 95% CIs were estimated using the Cox proportional hazards model. Other toxicities were summarized descriptively and analyzed using ordinal logistic regression or appropriate parametric or nonparametric tests. Longitudinal hematological changes were analyzed using mixed-design ANOVA. To account for multiple comparisons across secondary and exploratory outcomes and repeated time points, false discovery rate (FDR) adjustment was applied using the Benjamini-Hochberg procedure. Adjusted p-values are reported where applicable. The primary endpoint was not adjusted for multiplicity. All tests were two-sided, with *p* < 0.05 considered statistically significant.

### Reporting guidelines

This randomized controlled trial was reported in accordance with the Consolidated Standards of Reporting Trials (CONSORT) 2025 guidelines. A complete CONSORT checklist is provided as part of the supplementary materials file 2.

## Results

**Fig. 1:**
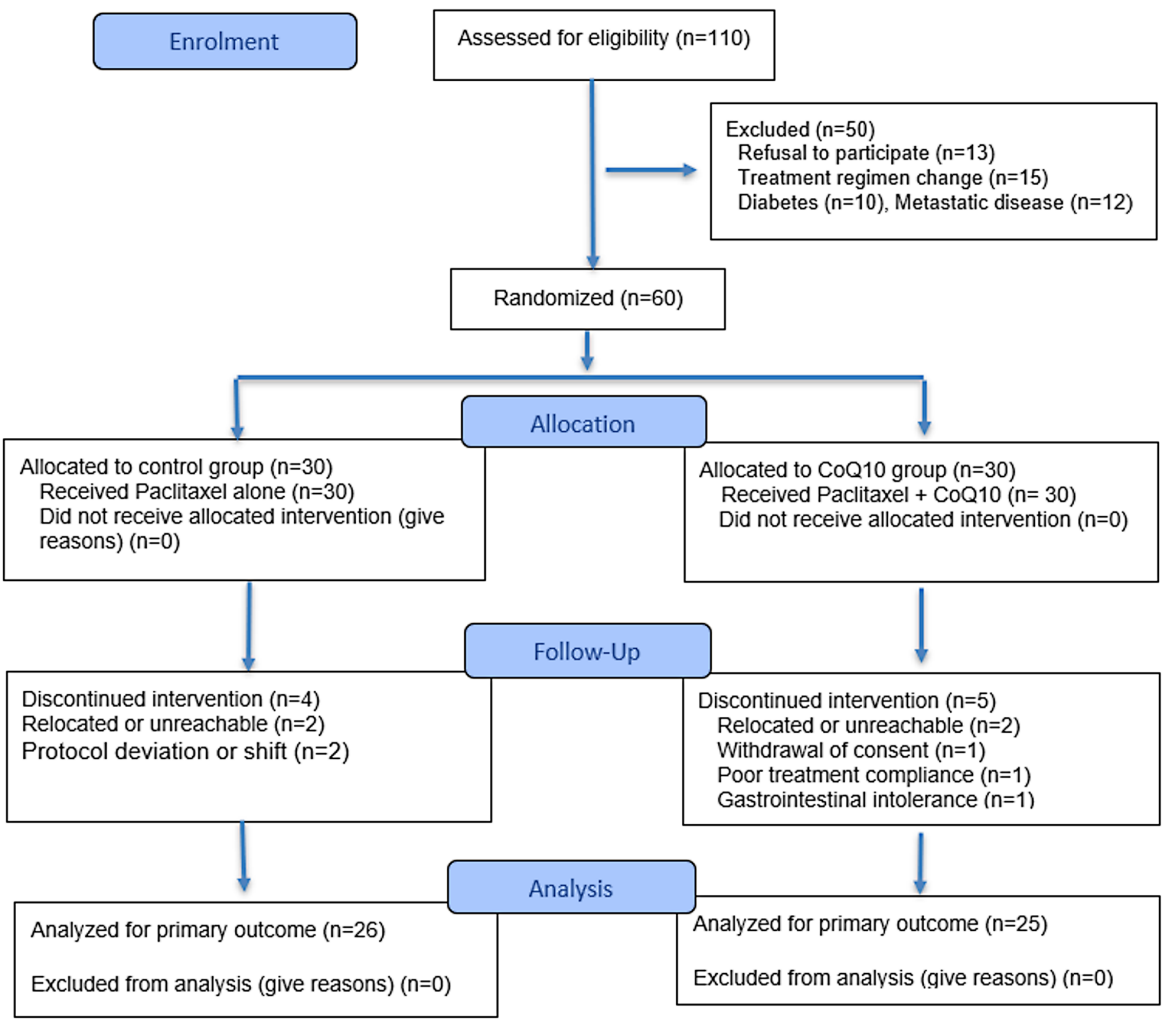
The Consolidated Standards of Reporting Trials (CONSORT) flow diagram showing patient enrollment, allocation, follow-up, and analysis

From October 2024 through March 2025, a total of 110 patients were assessed for eligibility. Fifty patients were excluded prior to randomization due to refusal to participate (*n* = 13), changes in chemotherapy regimen (*n* = 15), diabetes mellitus (*n* = 10), or the presence of metastatic disease (*n* = 12). The remaining 60 eligible patients were randomized in a 1:1 ratio into two groups: the CoQ10 group (*n* = 30), which received CoQ10 supplementation in addition to the standard paclitaxel-based chemotherapy regimen, and the control group (*n* = 30), who received the standard paclitaxel regimen alone. During the follow-up period, five patients in the CoQ10 group did not complete the study: two were lost to follow-up, one withdrew consent, one had poor adherence, and one experienced gastrointestinal intolerance. In the control group, four patients did not complete the study: two were lost to follow-up, and two switched to another chemotherapy protocol. Consequently, 25 patients in the CoQ10 group and 26 in the control group completed the study and were included in the per-protocol analysis. The study flow is shown in Fig. 1.

### Patients’ demographics

Baseline demographic and clinical characteristics were comparable between the control and CoQ10 groups (Table 1).

**Table 1:** Baseline clinical and demographic characteristics of the control and CoQ10 groups

Parameters	Control group (*n* = 26)	CoQ10 group (*n* = 25)	*p*-value
Age, mean ± SD (years)	51.12 ± 10.047	50.56 ± 12.376	0.861^a^
BMI, median (IQR) (Kg/m^2^)	29.30 (5.06)	27.60 (6.92)	0.692^b^
Menopausal state, n (%) Pre Post	8 (30.76) 18 (69.23)	14 (56.00) 11 (44.00)	0.069^c^
Contraception method history, n (%) None Hormonal Non-hormonal (IUD) Both	8 (30.76) 4 (15.38) 8 (30.76) 6 (23.07)	10 (40.00) 5 (20.00) 6 (24.00) 4 (16.00)	0.801^c^
Therapeutic Setting, n (%) Adjuvant Neoadjuvant	19 (73.07) 7 (26.92)	13 (52.00) 12 (48.00)	0.120^c^
Clinical prognostic stage, n (%) Stage I Stage IIA Stage IIB Stage IIIA Stage IIIB Stage IIIC	0 (0.00) 2 (7.69) 16 (61.54) 3 (11.54) 0 (0.00) 4 (15.38)	0 (0.00) 6 (24.00) 13 (52.00) 6 (24.00) 0 (0.00) 0 (0.00)	0.063^c^
ECOG-PS n, (%) 0 1 2	14 (53.84) 10 (38.46) 2 (7.69)	17 (68.00) 8 (32.00) 0 (0.00)	0.287^c^
Comorbidities, n (%) Hypertension Dyslipidemia	13 (50.00) 5 (19.23)	9 (36.00) 5 (20.00)	0.313^c^ 0.945^c^
Medications n, (%) Beta blocker CCBs Thiazide diuretics ACEIs ARBs Statins	4 (15.38) 6 (23.07) 9 (34.61) 2 (7.69) 8 (30.77) 5 (19.23)	5 (20.00) 3 (12.00) 5 (20.00) 1 (4.00) 4 (16.00) 5 (20.00)	0.666^c^ 0.300^c^ 0.242^c^ 0.575^c^ 0.214^c^ 0.945^c^
Cumulative dose of paclitaxel median (IQR) mg	1713.00 (146.00)	1680.00 (204.00)	0.098^b^

^a^ student t test, ^b^ Mann Whitney test, ^c^ Chi square test. *P*-value < 0.05 was considered statistically significant

No statistically significant differences were observed in age, BMI, menopausal status, therapeutic setting, clinical stage, ECOG performance status, comorbidities, or concomitant medications (*p* > 0.05).

Abbreviations: BMI, body mass index; IUD, intrauterine device; ECOG-PS, Eastern Cooperative Oncology Group performance status; CCBs, calcium channel blockers; ACEIs, angiotensin-converting enzyme inhibitors; ARBs, angiotensin receptor blockers.

### Paclitaxel-related adverse events

The cumulative incidence of adverse events is presented in Table 2. Cumulative incidence was defined as the proportion of patients who developed a specific adverse event of any grade at least once during the 12-week treatment period, calculated as the number of affected patients divided by the total number of patients in each group × 100. While Supplementary file 3 Table 1 reports the worst grade recorded per patient. The most frequently reported adverse events were mucositis, nausea, anemia, headache, insomnia, diarrhea, fatigue, and peripheral neuropathy. Compared with the control group, CoQ10 supplementation was associated with lower observed severity of several symptom-based adverse events, including peripheral neuropathy, fatigue, arthralgia, myalgia, headache, insomnia, mucositis, and diarrhea, particularly during the later weeks of treatment. No significant differences were observed between the two groups for nausea, vomiting, constipation, dry mouth, urinary tract pain, febrile neutropenia, or thrombocytopenia.

**Table 2:** Cumulative incidence of adverse events observed among the control group and CoQ10 group during 12 weeks of paclitaxel

	Control group (*n* = 26)					CoQ10 group *n*= (25)			
	Grade 1		Grade 2	Grade 3	Grade 4	Grade1	Grade 2	Grade 3	Grade 4
Nervous system disorders									
Peripheral neuropathy	20 (76.92)		25 (96.15)	6 (23.07)	0 (0.00)	22 (88.00)	17 (68.00)	3 (12.00)	0 (0.00)
Other nervous system disorders									
Headache	25 (96.15)		24 (92.30)	10 (38.46)	-	25 (100.00)	23 (92.00)	5 (20.00)	-
Insomnia	24 (92.30)		25 (96.15)	9 (34.61)	-	25 (100.00)	21 (84.00)	3 (12.00)	-
General disorders									
Fatigue	20 (76.92)		24 (92.30)	15 (57.69)	-	23 (92.00)	21 (84.00)	7 (28.00)	-
Musculoskeletal disorders									
Arthralgia	24 (92.30)		25 (96.15)	12 (46.15)	-	23 (92.00)	20 (80.00)	7 (28.00)	-
Myalgia	19 (73.07)		24 (92.30)	11 (42.30)	-	22 (88.00)	20 (80.00)	8 (32.00)	-
Gastrointestinal disorders									
Mucositis	26 (100.00)		9 (34.61)	2 (7.69)	0 (0.00)	25 (100.00)	7 (28.00)	0 (0.00)	0 (0.00)
Diarrhea	25 (96.15)		14 (53.84)	3 (11.53)	0 (0.00)	24 (96.00)	12 (48.00)	0 (0.00)	0 (0.00)
Dry mouth	25 (96.15)		5 (19.23)	0 (0.00)	-	24 (96.00)	6 (24.00)	0 (0.00)	-
Nausea	26 (100.00)		10 (38.46)	1 (3.84)	-	25 (100.00)	12 (48.00)	0 (0.00)	-
Vomiting	18 (69.23)		4 (15.38)	1 (3.84)	0 (0.00)	20 (80.00)	3 (12.00)	0 (0.00)	0 (0.00)
Constipation	9 (34.61)		7 (26.92)	0 (0.00)	0 (0.00)	7 (28.00)	4 (16.00)	0 (0.00)	0 (0.00)
Urinary tract pain	21 (53.84)		5 (19.23)	0 (0.00)	-	23 (92.00)	4 (16.00)	0 (0.00)	-
Hematological disorders									
Anemia	26 (100.00)		4 (15.38)	3(11.53)	0 (0.00)	24 (96.00)	5 (20.00)	0 (0.00)	0 (0.00)
Neutropenia	8 (30.76)		1 (3.84)	3 (11.53)	0 (0.00)	3 (12.00)	3 (12.00)	0 (0.00)	0 (0.00)
Thrombocytopenia	4 (15.38)		0 (0.00)	0 (0.00)	0 (0.00)	3 (12.00)	0 (0.00)	0 (0.00)	0 (0.00)
Febrile neutropenia	-		-	3 (11.53)	0 (0.00)	-	-	0 (0.00)	0 (0.00)

Grading was performed according to the National Cancer Institute Common Terminology Criteria for Adverse Events (CTCAE), version5.0. Data are expressed as number of patients (%). The control group included *n* = 26 participants, and the CoQ10 group included *n* = 25 participants

#### Effect of CoQ10 on peripheral neuropathy

The cumulative incidence of any-grade neuropathy was 100% in both groups. Clinically significant neuropathy (grade ≥ 2) occurred in 96% of patients in the control group versus 68% in the CoQ10 group, suggesting a lower observed risk in the CoQ10 arm (*p* = 0.01) (Table 3).

**Fig. 2:**
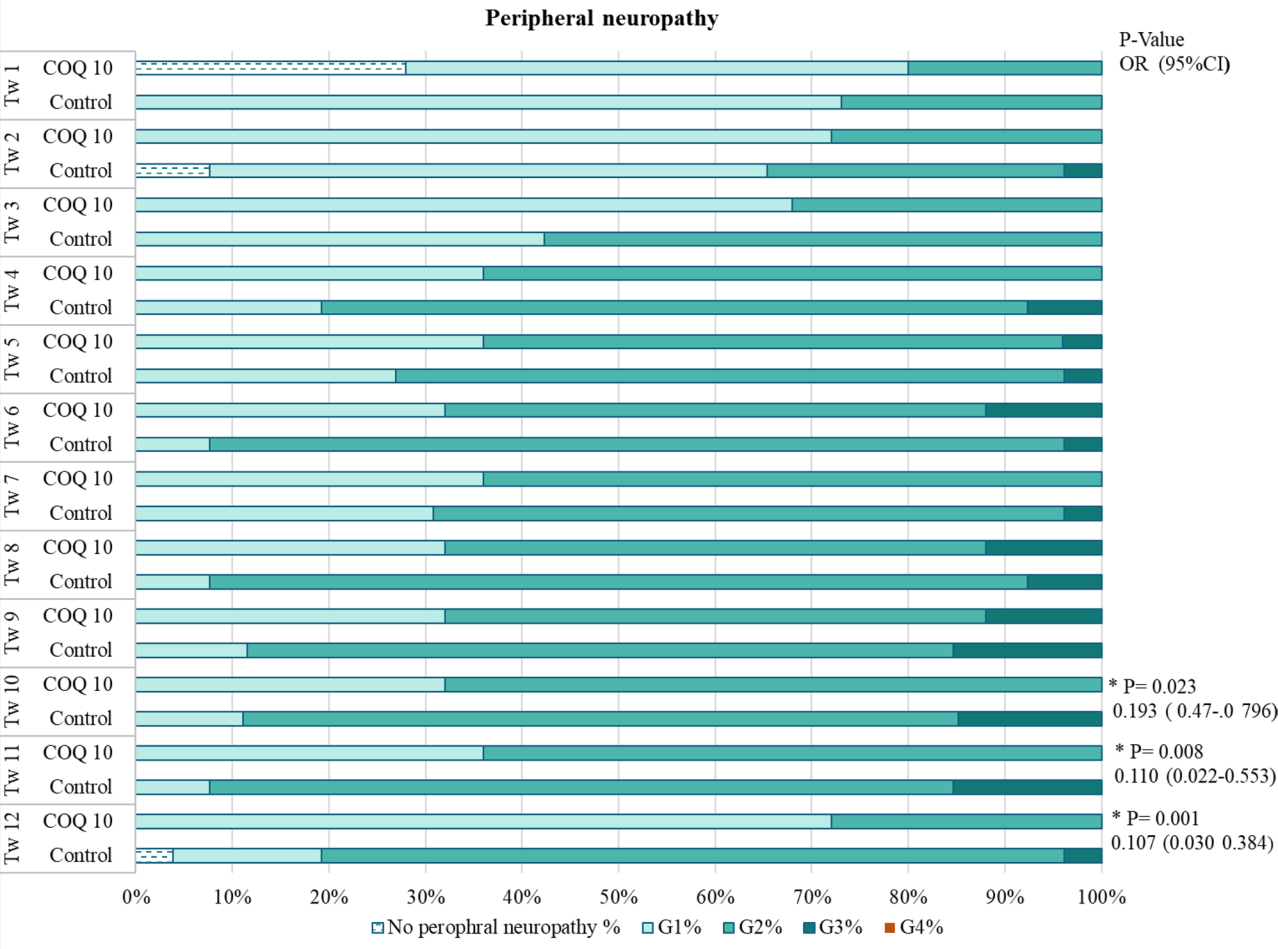
Incidence of peripheral neuropathy across CTCAE severity grades in CoQ10 and control groups during 12 weeks of paclitaxel therapy. Data is expressed as percentages of the frequency of events (CoQ10 group *n* = 25; control group *n* = 26). *C.I.* confidence interval, *G* severity grade, *OR* odds ratio, *Tw* weekly paclitaxel cycle. *Asterisk (*)* indicates a statistically significant difference (p-value < 0.05)

Longitudinal weekly analysis of any grade neuropathy (Fig. 2) showed that differences in neuropathy severity between groups became apparent in later treatment weeks. Specifically, the proportions of patients with grade 2–3 neuropathy in weeks 10, 11, and 12 were 76.9%, 92.3%, and 80.8% in the control group compared with 68.0%, 64.0%, and 28.0% in the CoQ10 group, respectively. These findings remained significant after false discovery rate (FDR) correction using the Benjamini-Hochberg procedure (adjusted *p* = 0.05, 0.04, and 0.02, respectively; Supplementary file 3 Tables 2 and 3).

**Fig. 3:**
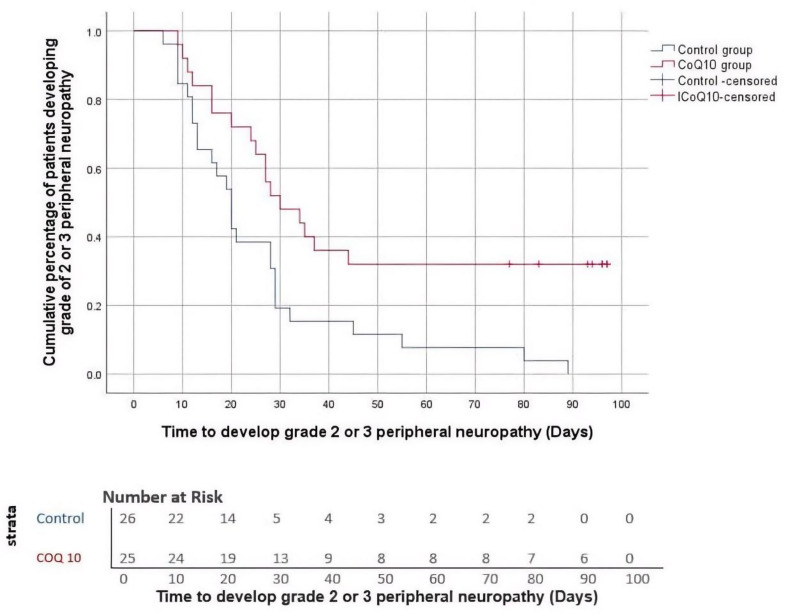
Kaplan-Meier curves showing time to onset of clinically significant peripheral neuropathy (grade ≥ 2, NCI-CTCAE v 5.0) in the CoQ10 and control groups. The red line represents the CoQ10 group; the blue line represents the control group

Time-to-onset analysis using Kaplan-Meier estimates demonstrated a significantly longer time to development of grade ≥ 2 peripheral neuropathy in the CoQ10 group compared with the control group. The median time to event was 30.0 days (95% CI: 18.6–41.4) in the CoQ10 group versus 20.0 days (95% CI: 16.3–23.7) in the control group (log-rank *p* = 0.005). A total of 26/26 patients in the control group and 17/25 patients in the CoQ10 group developed grade ≥ 2 neuropathy, while 8 patients in the CoQ10 group were censored. The Kaplan-Meier curve with censoring marks and the number-at-risk table is presented in Fig. 3, and the time-to-event summary is provided in Table 4. Full Kaplan-Meier estimates and hazard ratios are provided in Supplementary file 3 Table 4.

**Table 3:** Cumulative incidence of peripheral neuropathy during 12 weeks of paclitaxel treatment in control and CoQ10 groups

Group	*n*	Any-grade	Grade ≥ 2	OR (95% CI)	*p*-value
Control	26	100%	96%	-	-
CoQ10	25	100%	68%	0.08 (0.010–0.743)	0.01

**Table 4:** Summary of time to onset of grade ≥ 2 paclitaxel-induced peripheral neuropathy

Drug group	Median time to neuropathy (days)	95% CI (Lower-Upper)	Mean time (days)	95% CI (Lower-Upper)	Events / *n*	Censored (*n*)	Log-Rank *p*-value
Control (*n* = 26)	20.0	16.3–23.7	25.8	17.8–33.8	26 / 26	0	
CoQ10 (*n* = 25)	30.0	18.6–41.4	47.2	33.5–61.0	17 / 25	8	0.005

#### Effect of CoQ10 on insomnia and headache

**Fig. 4:**
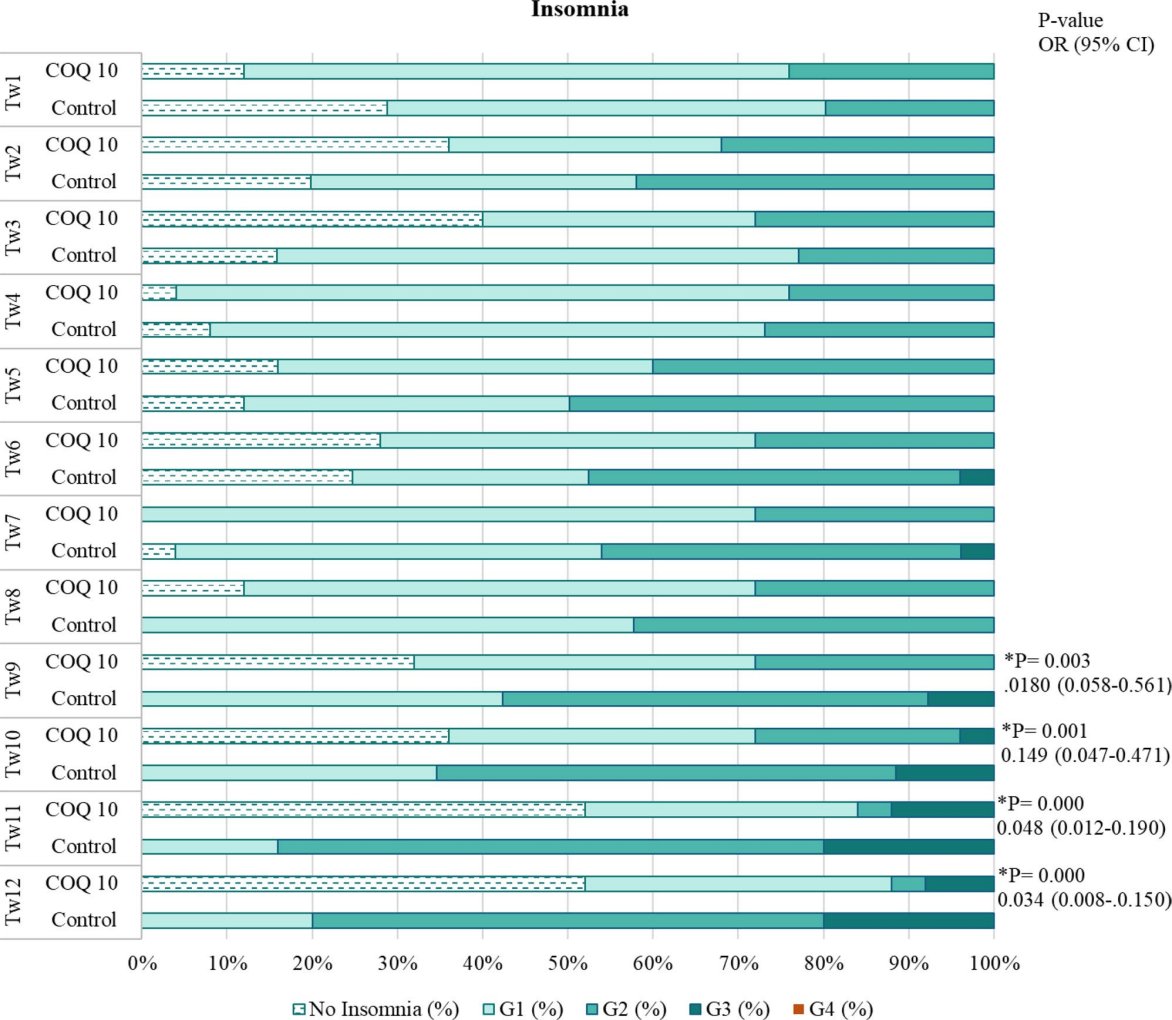
Incidence of insomnia across CTCAE severity grades in CoQ10 and control groups during 12 weeks of paclitaxel therapy. Data is expressed as percentages of the frequency of events (CoQ10 group *n* = 25; control group *n* = 26). *C.I.* confidence interval, *G* severity grade, *OR* odds ratio, *Tw* weekly paclitaxel cycle. *Asterisk (*)* indicates a statistically significant difference (p-value < 0.05)

**Fig. 5:**
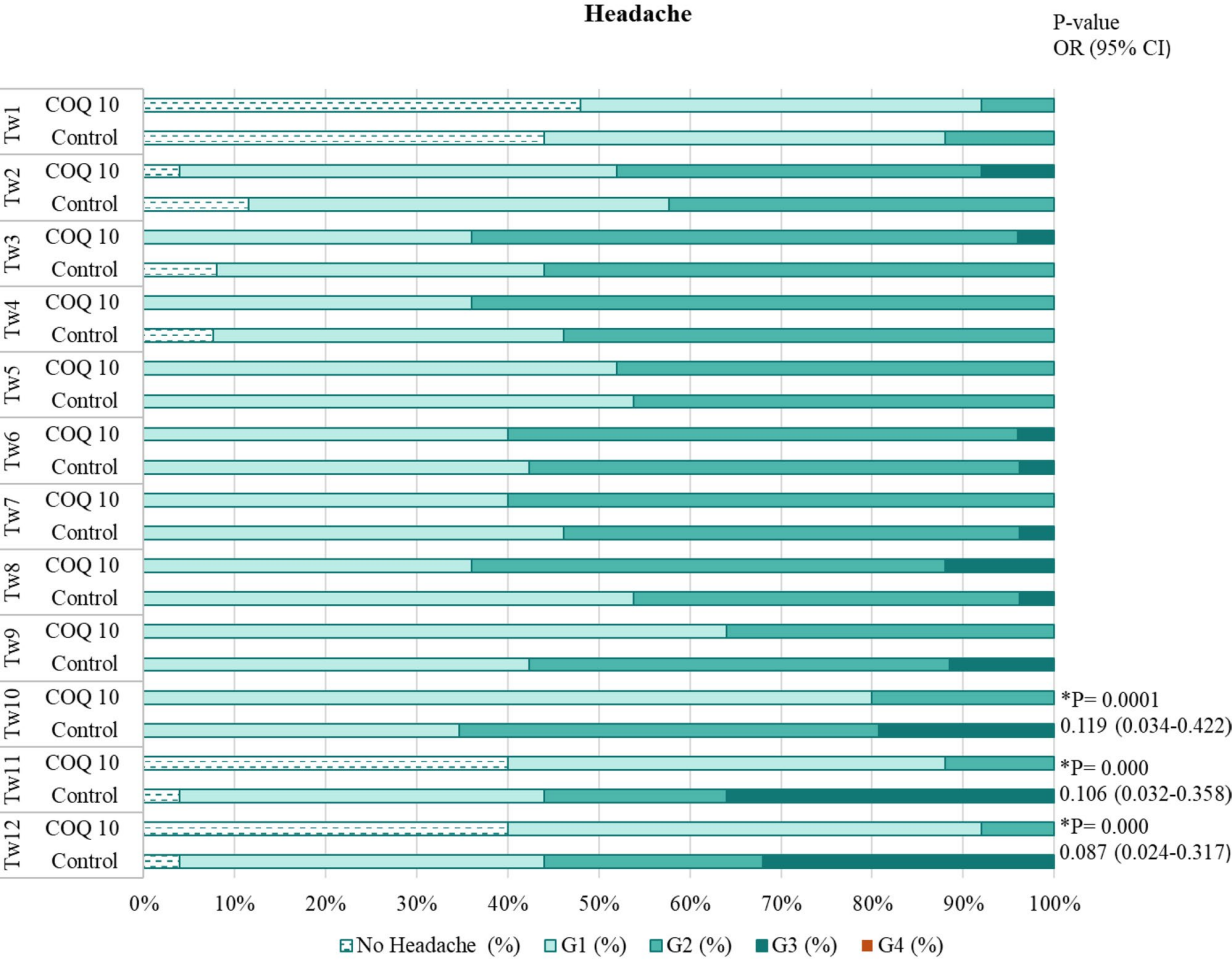
Incidence of headache across CTCAE severity grades in CoQ10 and control groups during 12 weeks of paclitaxel therapy. Data is expressed as percentages of the frequency of events (CoQ10 group *n* = 25; control group *n* = 26). *C.I.* confidence interval, *G* severity grade, *OR* odds ratio, *Tw* weekly paclitaxel cycle. *Asterisk (*)* indicates a statistically significant difference (p-value < 0.05)

Patients receiving CoQ10 showed lower observed severity of insomnia and headache compared with controls (Figs. 4 and 5). Differences in insomnia became apparent from week 9 and were most pronounced by week 12, when 76.9% of patients in the control group reported grade 2–3 insomnia versus 12% in the CoQ10 group (*p* < 0.001). Headache followed a similar pattern, with lower headache severity observed in the CoQ10 group from week 10 and persisting through weeks 11–12, during which grade 3 headache was observed in 30.8–34.6% of patients in the control group, compared with no patients in the CoQ10 group (*p* < 0.001). All significant between-group comparisons for insomnia and headache remained significant after FDR correction (adjusted p-values ranging from 0.01 to 0.03; Supplementary file 3 Table 2). Detailed weekly data are provided in supplementary file 3 Tables 5 and 6.

#### Effect of CoQ10 on fatigue

**Fig. 6:**
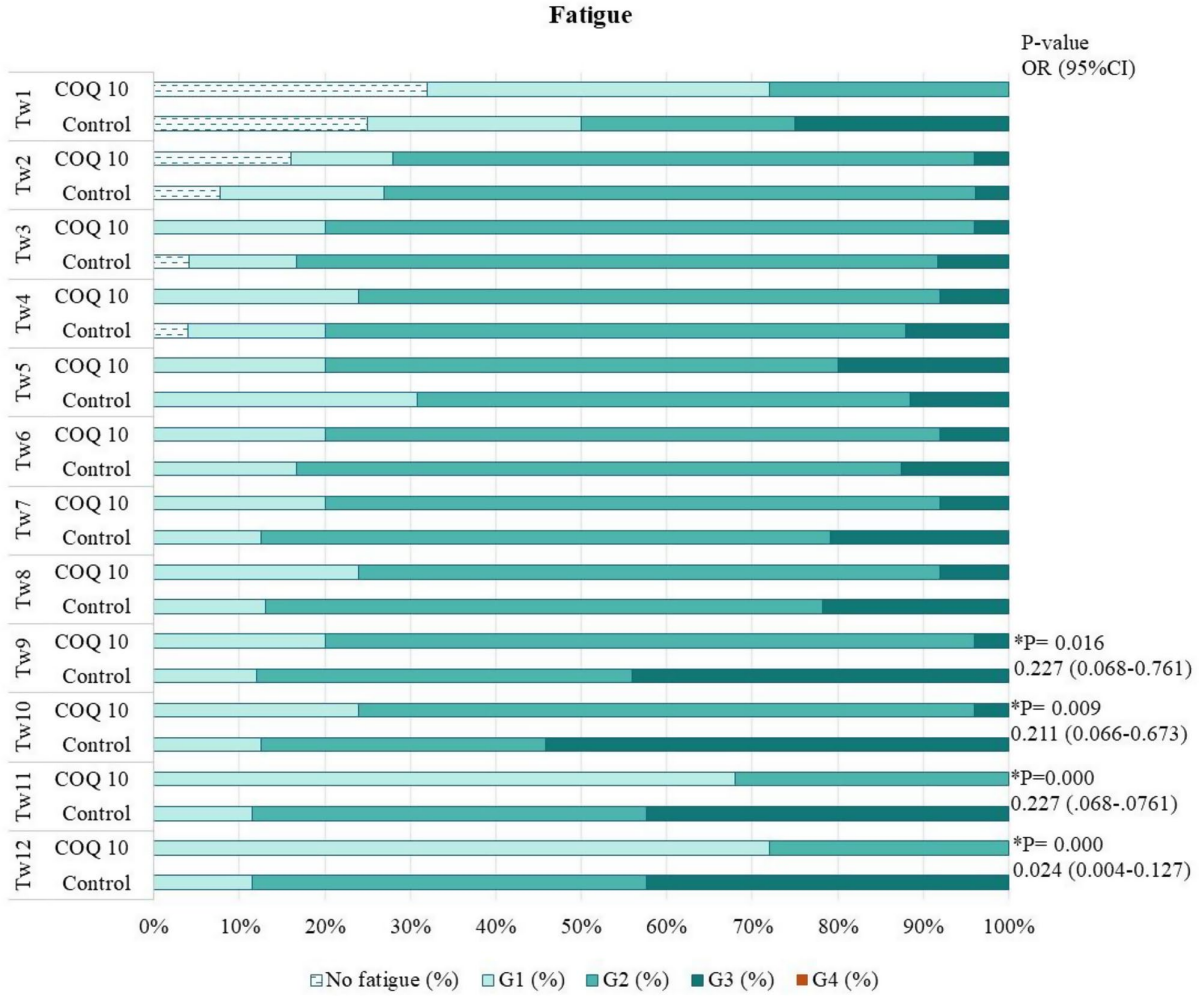
Incidence of fatigue across CTCAE severity grades in CoQ10 and control groups during 12 weeks of paclitaxel therapy. Data is expressed as percentages of the frequency of events (CoQ10 group *n* = 25; control group *n* = 26). *C.I.* confidence interval, *G* severity grade, *OR* odds ratio, *Tw* weekly paclitaxel cycle. *Asterisk (*)* indicates a statistically significant difference (p-value < 0.05)

CoQ10 supplementation was associated with lower observed severity of fatigue beginning at week 9 (Fig. 6). At this time, 42.3% of control patients experienced grade 3 fatigue compared with 4% in the CoQ10 group (*p* = 0.016). This between-group difference became more pronounced over subsequent weeks, 46.2% of control patients still reported grade 3 fatigue, no patients in the CoQ10 group had fatigue above grade 2. These differences also remained significant following FDR adjustment (adjusted p-values = 0.04, 0.04, and 0.01 for weeks 9–11, respectively are reported in supplementary file 3 Table 2. Detailed weekly data are provided in Supplementary file 3 Table 7.

#### Effect of CoQ10 on arthralgia and myalgia

**Fig. 7:**
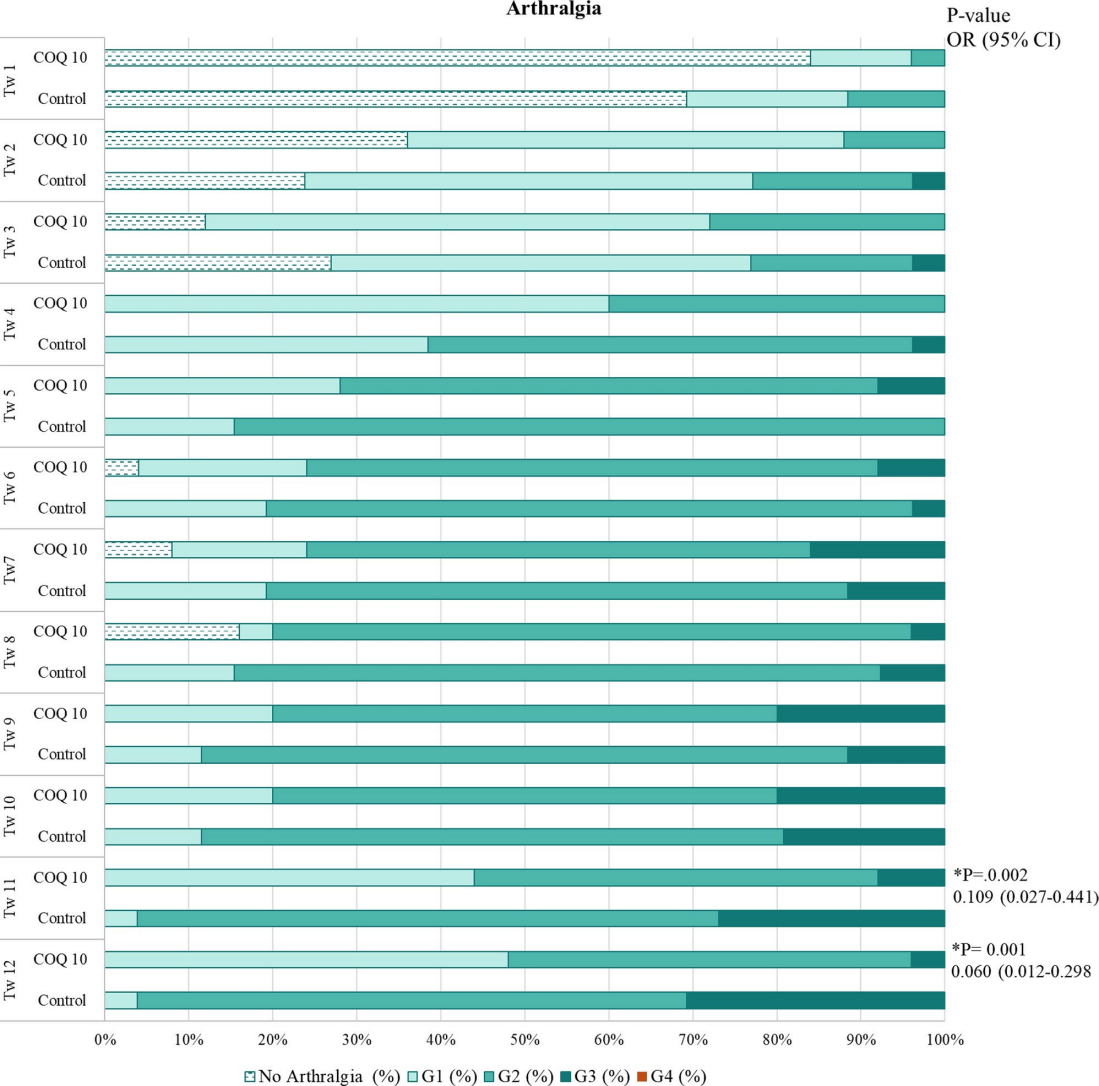
Incidence of arthralgia across CTCAE severity grades in CoQ10 and control groups during 12 weeks of paclitaxel therapy. Data is expressed as percentages of the frequency of events (CoQ10 group *n* = 25; control group *n* = 26). *C.I.* confidence interval, *G* severity grade, *OR* odds ratio, *Tw* weekly paclitaxel cycle. *Asterisk (*)* indicates a statistically significant difference (p-value < 0.05)

**Fig. 8:**
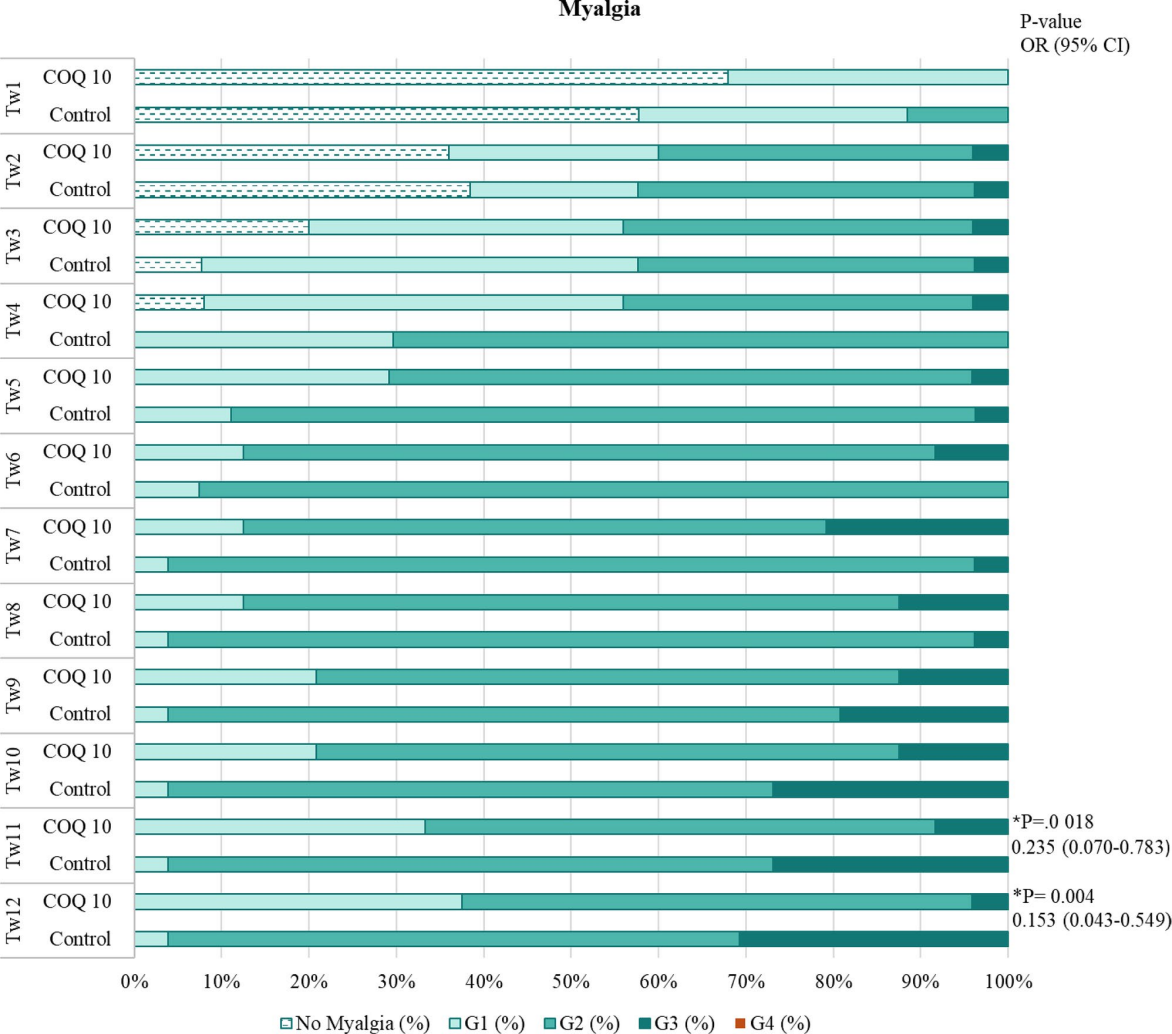
Incidence of myalgia across CTCAE severity grades in CoQ10 and control groups during 12 weeks of paclitaxel therapy. Data is expressed as percentages of the frequency of events (CoQ10 group *n* = 25; control group *n* = 26). *C.I.* confidence interval, *G* severity grade, *OR* odds ratio, *Tw* weekly paclitaxel cycle. *Asterisk (*)* indicates a statistically significant difference (p-value < 0.05)

Both arthralgia and myalgia exhibited a similar pattern throughout paclitaxel treatment, as illustrated in Figs. 7 and 8, respectively. Significant between-group differences emerged during the final two weeks of treatment. By week 12, 30.8% of patients in the control group experienced grade 3 arthralgia, compared with 4.0% in the CoQ10 group (*p* = 0.001). Likewise, severe myalgia (grade 3) was reported in 30.8% of controls versus 8% in the CoQ10 group (*p* = 0.004). The observed differences remained statistically significant after FDR correction (adjusted p-values ranging from 0.02 to 0.05; Supplementary file 3 Table 2). Detailed weekly data are provided in Supplementary file 3 Tables 8 and 9.

#### Gastrointestinal and urinary adverse events

**Fig. 9:**
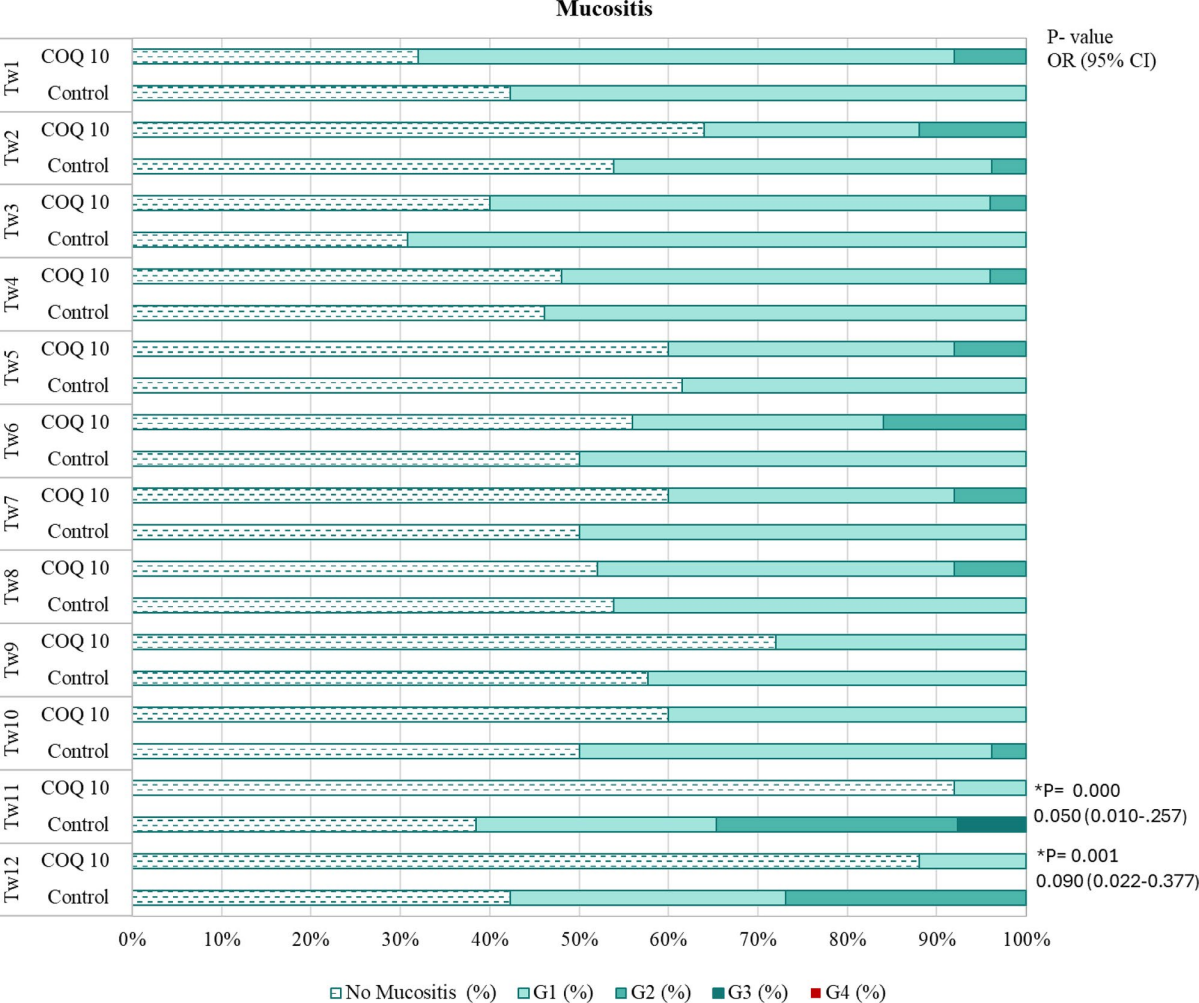
Incidence of oral mucositis across CTCAE severity grades in CoQ10 and control groups during 12 weeks of paclitaxel therapy. Data is expressed as percentages of the frequency of events (CoQ10 group *n* = 25; control group *n* = 26). *C.I.* confidence interval, *G* severity grade, *OR* odds ratio, *Tw* weekly paclitaxel cycle. *Asterisk (*)* indicates a statistically significant difference (p-value < 0.05)

**Fig. 10:**
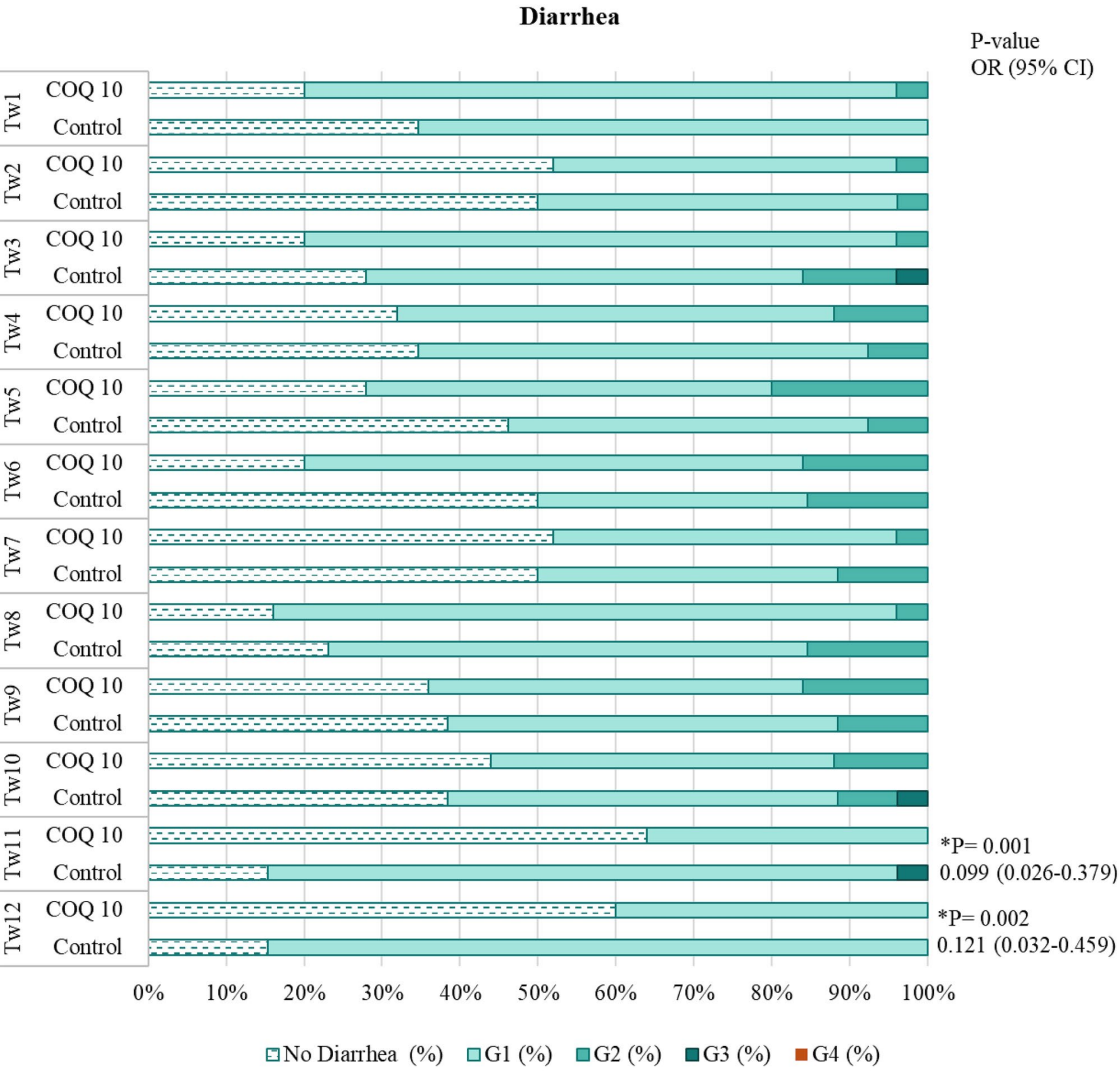
Incidence of diarrhea across CTCAE severity grades in CoQ10 and control groups during 12 weeks of paclitaxel therapy. Data is expressed as percentages of the frequency of events (CoQ10 group *n* = 25; control group *n* = 26). *C.I.* confidence interval, *G* severity grade, *OR* odds ratio, *Tw* weekly paclitaxel cycle. *Asterisk (*)* indicates a statistically significant difference (p-value < 0.05)

As illustrated in Figs. 9 and 10, significant between-group differences were observed for oral mucositis and diarrhea during weeks 11 and 12. At week 11, 34.6% of patients in the control group experienced grade 2–3 mucositis, while no cases were observed in the CoQ10 group (*p* < 0.001). By week 12, 57.7% of the control group experienced grade 2–3 mucositis compared with no patients in the CoQ10-treated group (*p* = 0.001). A similar pattern was observed for diarrhea, with diarrhea reported in 84.6% of patients in the control group at week 12 (*p* = 0.002). Weekly progression details are presented in Tables 10 and 11 (Supplementary Material). These findings remained significant after FDR correction, with adjusted p-values ranging from 0.01 to 0.03 (Supplementary file 3 Table 2). The remaining gastrointestinal adverse events, including constipation, dry mouth, nausea, vomiting, and urinary tract pain-occurred at comparable frequencies and severity grades, with no statistically significant differences between groups; Supplementary file 3 Tables 10 and 11for further details.

#### Effect of CoQ10 on dermatological toxicities

At baseline, both groups showed a similar distribution of nail changes (*p* = 0.428) (Table 5). However, after 12 weeks of paclitaxel, patients receiving CoQ10 were more likely to maintain nail plate integrity (44.0% vs. 11.5%), while nail changes were more frequent in the control group (88.5% vs. 56.0%). This difference was statistically significant with (*p* = 0.009). The association remained significant after FDR correction (adjusted *p* = 0.04). At baseline, there were no differences between the groups in alopecia grades (*p* = 0.428). At 12th week, fewer patients in the CoQ10 group experienced severe (grade 2) alopecia compared with the control group (56.0% vs. 88.5%), while a higher proportion had grade 1 alopecia (44.0% vs. 11.5%). Although this difference showed a numerical trend favoring CoQ10, it did not reach statistical significance (*p* = 0.329) as shown Supplementary file 3 Table 17.

#### Effect on hemoglobin and anemia severity

**Fig. 11:**
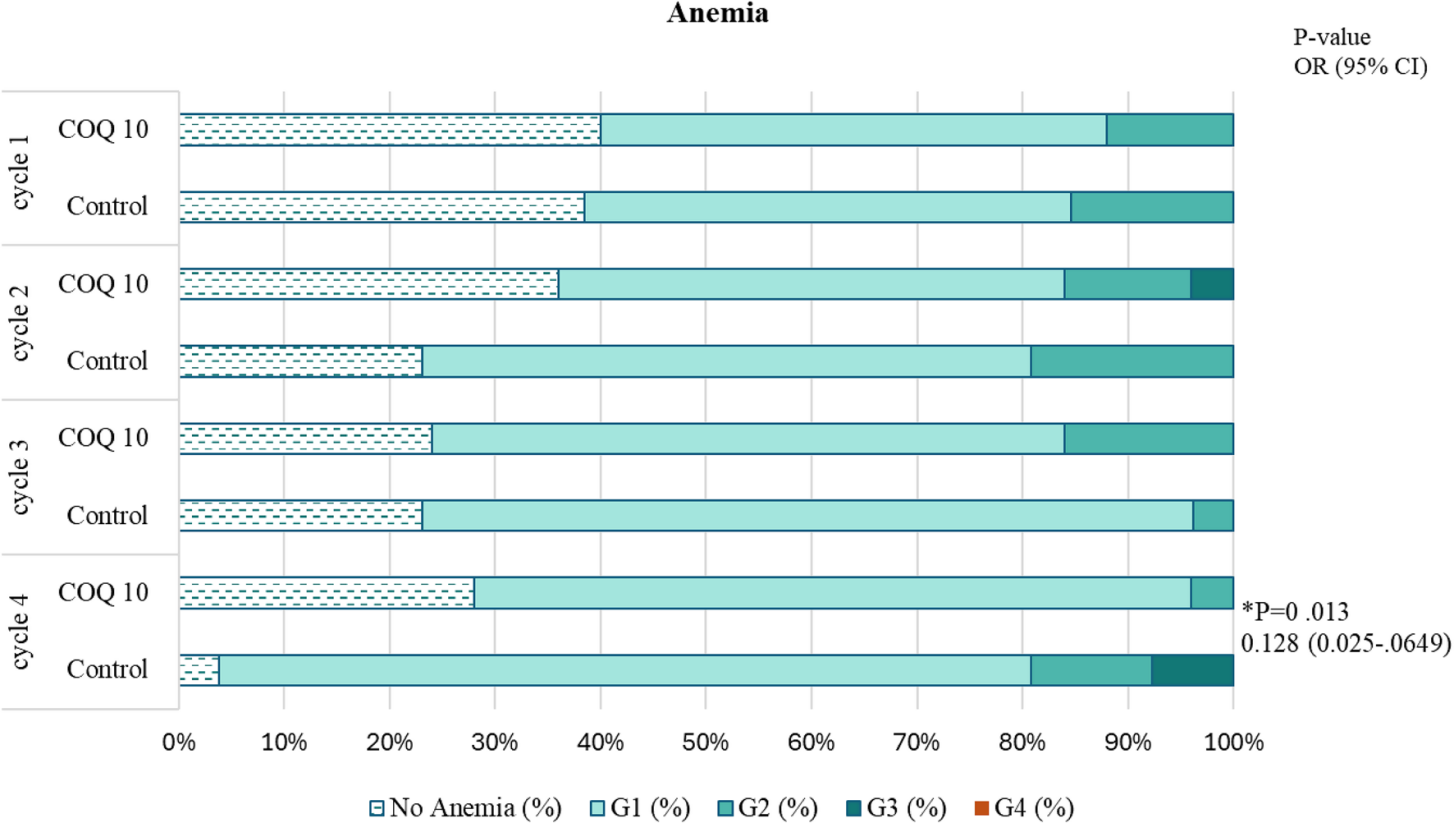
Incidence of anemia across CTCAE severity grades in CoQ10 and control groups during 12 weeks of paclitaxel therapy. Data is expressed as percentages of the frequency of events (CoQ10 group *n* = 25; control group *n* = 26). *C.I.* confidence interval, *G* severity grade, *OR* odds ratio, *Tw* weekly paclitaxel cycle. *Asterisk (*)* indicates a statistically significant difference (p-value < 0.05)

**Fig. 12:**
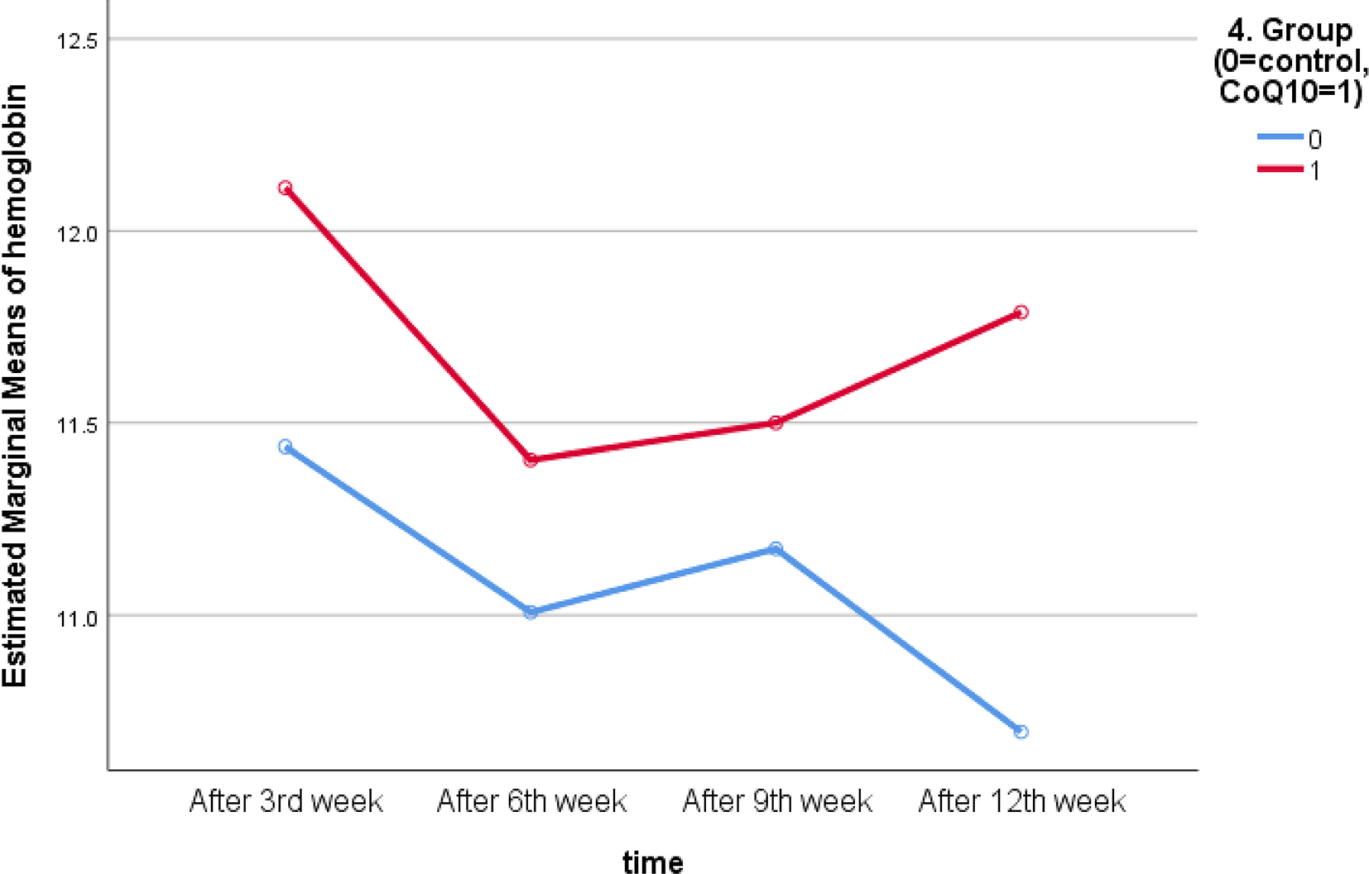
Longitudinal changes in hemoglobin levels across chemotherapy cycles in the control and CoQ10 groups

Hemoglobin levels were comparable between the CoQ10 and control groups and remained comparable through weeks 3 and 6 of paclitaxel administration. However, at the 12th week, the CoQ10 group had significantly higher hemoglobin values (11.79 ± 1.31 g/dL) compared to the control group (10.70 ± 1.54 g/dL; p = 0.009) Table 6. This difference also remained significant after FDR correction (adjusted p = 0.04). This finding was consistent with clinical anemia grading (Fig. 11), which showed a significant between-group difference at week 12 (p = 0.013), with fewer patients progressing to grade 2–3 anemia in the CoQ10 group (OR = 0.128), where fewer patients progress to grades 2–3 in the CoQ10 group (OR = 0.128). A mixed-design ANOVA was used to assess the interaction between time and treatment, yielding a significant interaction effect (p = 0.002; Fig. 12). Further details of anemia severity grades are provided in (supplementary material file 3 Table 18).

**Table 5:** Frequencies of CTCAE severity grades of nail changes among the control group and CoQ10 group

Characteristic	Control group (*n* = 26)	CoQ10 group (*n* = 25)	*p*-value	FDR significance
**At Baseline**				
No changes	7 (26.9%)	9 (36.0%)	0.428^a^	-
grade1	19 (73.1%)	16 (64.0%)		
**At 12th week**				
No changes	3 (11.5%%)	11 (44.0%%)	**0.009** ^**a**^	**0.04**
grade1	23 (88.5%%)	14(56.0%%)		
^a^ Chi-square test.				
A p-value < 0.05 was considered statistically significant.				

**Table 6:** Comparison of hemoglobin levels between the control group and CoQ10 group

Time point (Mean ± SD)	Control group (*n* = 26)	CoQ10 group (*n* = 25)	*p*-value	FDR significance
At 3rd week	11.438 ± 1.191	12.112 ± 1.326	0.062^a^	-
At 6th week	11.007 ± 1.248	11.404 ± 1.411	0.293^a^	-
At 9th week	11.173 ± 0.720	11.500 ± 1.523	0.329^a^	-
At 12th week	10.696 ± 1.541	11.788 ± 1.308	**0.009** ^**a**^	**0.04**

^a^ Student t testA p-value < 0.05 was considered statistically significant.

#### Effect of other hematological AEs

Patients in the CoQ10 group showed a numerical trend towards a lower incidence and severity of neutropenia, febrile neutropenia, and thrombocytopenia, although these differences were not statistically significant compared to the control group. Further details are provided in Supplementary file 3 Tables 19, 20 and 21.

#### Effect on ejection fraction

Left ventricular ejection fraction values are summarized in Table 7. At baseline, there was no statistically significant difference between the control group (64.31 ± 2.26%) and the CoQ10 group (64.80 ± 2.55%) with (*p* = 0.468). At 12th week of paclitaxel, ejection fraction was significantly higher in the CoQ10 group (66.32 ± 3.02%) compared with the control group (63.81 ± 3.02%; *p* = 0.005). The between-group difference after week 12 remained significant following FDR adjustment (adjusted *p* = 0.03). Furthermore, within-group analysis showed a significant increase in the CoQ10 group from 64.80 ± 2.55% at baseline to 66.32.

± 3.02% post-treatment (*p* = 0.008), while the change in the control group from 64.31 ± 2.26% to 63.81 ± 3.02% was not statistically significant (*p* = 0.362).

**Table 7:** Comparison of ejection fraction between the CoQ10 and control groups at baseline and after paclitaxel treatment

Group (Mean ± SD)	Control group (*n* = 26)	CoQ10 group (*n* = 25)	P1-value	FDR significance
At baseline	64.31 ± 2.259	64.80 ± 2.550	0.468^a^	-
After 12th week	63.81 ± 3.020	66.32 ± 3.02	0.005^a^	0.03
P2 value within group	0.362^b^	0.008^b^		

^a^ Student t test, ^b^ paired t testP1: difference among groups. P2: difference within group.A p-value < 0.05 was considered statistically significant.

To provide a comprehensive overview of CoQ10-associated findings across multiple toxicity endpoints and robustness analyses, Supplementary file 3 Table 22, and 23 summarize comparisons modified intention-to-treat and per-protocol analyses, sensitivity analyses, and overall robustness assessments of the observed outcomes.

### Adherence, tolerability, and follow-up

CoQ10 supplementation was well tolerated overall. One patient developed mild gastrointestinal discomfort and withdrew during week 3. No other CoQ10-related adverse events, including nausea, vomiting, diarrhea, dizziness, photophobia, irritability, headache, or insomnia, were reported among the remaining participants. Adherence was high among completers. Of the 25 patients who completed the 12-week intervention, 24 (96.0%) achieved the predefined adherence threshold of ≥ 90% of prescribed doses based on pill counts and dosing diaries; one patient did not meet the adherence criterion due to poor adherence and withdrawal. Follow-up was limited to the 12-week treatment period and was not designed to assess tumor response and pathological complete response (pCR).

## Discussion

Paclitaxel remains a cornerstone of chemotherapy for breast cancer; however, its clinical utility is often limited by treatment-related toxicities that negatively affect patients’ quality of life and adherence to therapy. In the present study, supplementation with CoQ10 was associated with a reduced incidence or severity of several paclitaxel-related adverse effects, including peripheral neuropathy, arthralgia, myalgia, mucositis, diarrhea, and anemia, along with lower observed severity of fatigue, insomnia, and headache, and favorable changes in left ventricular ejection fraction. These findings suggest a potential supportive role for CoQ10 in mitigating treatment-related toxicities in patients receiving paclitaxel.

In this study, differences between groups emerged gradually, with lower observed severity of fatigue and insomnia from week 9, neuropathy and headache from week 10, and mucositis, diarrhea, arthralgia, and myalgia from week 11. This delayed pattern may be related to the pharmacological profile of CoQ10 as a fat-soluble compound that requires time to accumulate in tissues [56, 57]. As paclitaxel toxicities arise cumulatively across treatment cycles, CoQ10’s potential protective effects became clinically evident once adequate tissue saturation and cellular repair were achieved, a pattern consistent with prior evidence showing stronger effects after several weeks of supplementation [58, 59].

Paclitaxel-induced peripheral neuropathy is one of the most common and clinically significant dose-limiting toxicities associated with this agent [60, 61]. In the present study, CoQ10 supplementation was associated with a significantly lower cumulative incidence of grade ≥ 2 neuropathy over 12 weeks of paclitaxel treatment compared with the control group. In addition, Kaplan-Meier analysis demonstrated a delayed onset of grade ≥ 2 neuropathy of approximately 10 days, suggesting a potential association with delayed neuropathy onset. Mechanistically, CoQ10 may exert neuroprotective effects through its role in mitochondrial electron transport, enhancement of ATP production, and reduction of oxidative stress, all of which are implicated in chemotherapy-induced neuronal injury [52]. While direct clinical evidence in CIPN remains limited, supportive data from other neuropathic conditions suggests a potential role in neuropathic symptom modulation. For example, adjunctive CoQ10 has been associated with improvement in symptoms severity in diabetic peripheral neuropathy, including reductions in pain and functional impairment [62, 63]. Although the pathophysiology of diabetic neuropathy differs from chemotherapy-induced neurotoxicity, both conditions involve oxidative stress and mitochondrial dysfunction, which provides a plausible biological overlap supporting the observed findings [64]. It should be noted that in the worst-case sensitivity analysis, the difference in peripheral neuropathy at week 10 did not retain statistical significance, suggesting that the observed effect may be influenced by missing data assumptions. Therefore, these findings should be interpreted as exploratory and require confirmation in larger, adequately powered randomized trials.

In addition to its impact on neuropathy, daily supplementation with 400 mg of CoQ10 for three months was also associated with lower headache severity, as reflected by lower headache severity compared with the control group. Although migraine differs clinically from chemotherapy-related headache, previous studies have reported that CoQ10 supplementation may reduce migraine frequency, duration, and associated symptoms, including nausea [65–70]. Furthermore, Benefits have also been reported with CoQ10 doses ranging from 100 to 300 mg/day, with improvements in headache severity observed in both migraine and fibromyalgia patients [71–73].

Fatigue is another common and debilitating adverse event that markedly impairs patients’ quality of life (QoL) [11]. In the current study, patients in the CoQ10 group predominantly experienced grade 1 fatigue, a considerable proportion of patients in the control group experienced grade 3 fatigue. To our knowledge, this represents the first clinical investigation of CoQ10 in breast cancer patients receiving paclitaxel exclusively. Evidence from other clinical conditions, such as fibromyalgia and chronic fatigue syndrome, also suggests a potential benefit of CoQ10 in fatigue-related symptoms [71–74].

The anti-fatigue effects of CoQ10 in this study were accompanied by lower insomnia severity, with fewer patients experiencing moderate-to-severe insomnia. Supportive observations from non-oncology populations, including individuals with work-related fatigue and chronic fatigue syndromes, have reported improvements in sleep duration and quality with CoQ10 supplementation, providing indirect supportive context for the present findings [72, 75, 76]. These observations suggest that CoQ10 may have a supportive role in reducing chemotherapy-related fatigue and insomnia symptoms, although confirmation in trials using validated fatigue and sleep instruments is warranted.

CoQ10 supplementation was also associated with lower observed severity of paclitaxel-induced arthralgia and myalgia. This clinical benefit is consistent with previous observations that breast cancer patients often have reduced endogenous CoQ10 levels, which could increase their susceptibility to musculoskeletal toxicity [13]. Similarly, statin therapy has been associated with lower CoQ10 levels and increased muscle symptoms; importantly, CoQ10 supplementation has been investigated in statin-associated muscle symptoms, with some studies reporting reductions in muscle pain severity [77–79]. These findings provide indirect biological plausibility for the observed association between CoQ10 supplementation and lower musculoskeletal symptom severity in the present study.

Additional indirect supportive evidence for CoQ10 has been reported in musculoskeletal conditions. In osteoarthritis, higher CoQ10 levels have been associated with improved muscle performance parameters, while in rheumatoid arthritis, supplementation has been linked to reductions in pain and disease activity [80, 81]. In the present study, CoQ10 supplementation was associated with lower observed severity of paclitaxel-induced musculoskeletal symptoms, with significant between-group differences emerging during later treatment cycles.

Furthermore, CoQ10 supplementation also may extend its benefits to gastrointestinal toxicity, as reflected by lower severity grades of oral mucositis and diarrhea. To date, no previous study appears to have directly evaluated CoQ10 supplementation in chemotherapy-related gastrointestinal toxicity. Supportive mechanistic evidence from inflammatory conditions of the oral cavity has suggested a role for CoQ10 in modulating tissue inflammation and oxidative stress, which may contribute to improved periodontal health [82–84]. In the present study, CoQ10 supplementation was also associated with lower diarrhea severity compared with the control group. This observation is supported by an earlier study reporting that ubiquinol reduced defecation frequency in patients with functional bowel disorders [85].

Dermatological toxicities are among the most distressing and psychologically impactful adverse effects of chemotherapy, particularly for women, given their implications for body image, social interaction, and quality of life [86]. Although CoQ10 supplementation was not significantly associated with improvement in alopecia, it was associated with lower observed nail toxicity. A higher proportion of patients in the CoQ10 group maintained normal nail appearance, whereas nail ridging and discoloration were more common in the control group. These findings are supported by mechanistic and experimental data suggesting a role for CoQ10 in keratinocyte function, epidermal integrity, and dermal repair processes [87], as well as previous clinical observations reporting improved nail mechanical properties with supplementation [88]. Together, these findings may suggest a potential association between CoQ10 supplementation and lower chemotherapy-induced nail toxicity.

In the current study, CoQ10 supplementation was associated with preservation of hemoglobin levels during paclitaxel therapy, with the CoQ10 group maintaining higher and more stable hemoglobin values by the fourth cycle and a lower incidence of moderate-to-severe anemia. Although CoQ10 has not yet been investigated in cancer-related anemia, hematologic improvements have been reported with CoQ10 supplementation in other clinical contexts. Supportive evidence includes reports of improved hemoglobin levels in mitochondrial sideroblastic anemia, reduced exercise-related declines in red blood cell indices in healthy individuals, and improved hematologic parameters in sickle cell disease following CoQ10 supplementation [89–91]. Overall, these observations suggest a potential role for CoQ10 in supporting hematologic stability during chemotherapy; however, this hypothesis requires confirmation in studies specifically designed to evaluate chemotherapy-related anemia.

A notable exploratory finding of this study was the observed difference in left ventricular ejection fraction between patients receiving CoQ10 supplementation and those in the control group after 12 weeks of paclitaxel therapy following prior exposure to doxorubicin. Patients in the CoQ10 group demonstrated a modest increase in ejection fraction compared with baseline and with the control group at the end of treatment, suggesting a potentially favorable effect on cardiac function in this clinical context. Preclinical studies have proposed that CoQ10 may support mitochondrial function, reduce oxidative stress, and improve myocardial energy metabolism, which could theoretically contribute to preservation of myocardial function under conditions of chemotherapy-induced stress [48, 92]. In addition, previous clinical reports have explored CoQ10 use in patients receiving anthracyclines and have suggested maintenance of cardiac function parameters, although evidence remains inconsistent and derived from heterogeneous study designs [47, 92, 93]. However, these findings should be interpreted with caution. Cardiac assessment in the present study was limited to baseline and post-treatment echocardiography, and the study was not specifically powered to evaluate cardiotoxicity or long-term cardiac outcomes. Therefore, the observed differences should be considered exploratory and hypothesis-generating rather than confirmatory evidence of a cardioprotective effect. Further adequately powered studies with serial cardiac imaging and longer follow-up are required to clarify the potential role of CoQ10 in preserving left ventricular function during chemotherapy.

CoQ10 supplementation was generally well tolerated, with only one patient discontinuing treatment due to mild gastrointestinal discomfort. No serious adverse events related to CoQ10 were observed. Given the theoretical concern that antioxidants could interfere with chemotherapy efficacy, available clinical and preclinical evidence has not consistently demonstrated a reduction in chemotherapy effectiveness. Clinical studies have not reported worsened oncologic outcomes with CoQ10 supplementation [94]. Preclinical and external clinical studies suggest that CoQ10-related modulation of cellular redox balance has not consistently been associated with impaired chemotherapy activity in available studies; however, its potential impact on tumor response or interaction with cytotoxic therapy was not directly evaluated in the present study [95–98].

### Strengths and limitations

Key strengths of this study include the use of standardized NCI-CTCAE v5.0 criteria for toxicity assessment and the comprehensive evaluation of both subjective and objective outcomes. Rigorous follow-up, combining face-to-face assessments with structured telephone monitoring, supported systematic documentation of adverse events. To our knowledge, this is the first clinical trial to evaluate CoQ10 supplementation across multiple paclitaxel-related toxicities, and its favorable safety and tolerability support its feasibility as a supportive care strategy. Several limitations should be acknowledged. The open-label design may introduce some degree of observer and patient-expectation bias, particularly for subjective, symptom-based endpoints such as neuropathy, fatigue, insomnia, headache, arthralgia, myalgia, mucositis, and diarrhea. The use of standardized CTCAE-based grading and structured assessment methods was intended to enhance consistency in outcome evaluation. Accordingly, findings related to subjective endpoints should be interpreted with appropriate caution. In addition, validated patient-reported outcome instruments were not incorporated, and symptom assessment relied on clinician-rated evaluations, which may limit direct comparability with studies using formal PRO tools. Although baseline characteristics did not differ significantly between groups, a numerical imbalance was observed in the proportion of patients receiving therapy in the neoadjuvant setting. Given the modest sample size (*n* = 51), residual confounding from this imbalance cannot be fully excluded. Therefore, any inference regarding the interaction between CoQ10 and chemotherapy efficacy or tumor response should be interpreted with caution and requires confirmation in adequately powered, prospectively designed trials with predefined oncologic endpoints. The follow-up duration was limited to 12 weeks and did not permit assessment of long-term outcomes such as persistent neuropathy, disease recurrence, or survival. Finally, while the sample size was adequate for the primary endpoint, secondary outcomes were not individually powered and should be interpreted as exploratory. Larger, multicenter, preferably blinded studies incorporating validated patient-reported outcome measures and extended follow-up are warranted to confirm and expand upon these findings.

## Conclusion

In this small open-label study, CoQ10 supplementation was associated with lower paclitaxel-related toxicity, including lower observed incidence or severity of neuropathy, fatigue, insomnia, arthralgia, myalgia, mucositis, diarrhea, and nail changes. In addition, better preservation of hemoglobin levels and favorable changes in left ventricular ejection fraction were observed. These findings suggest that CoQ10 may represent a potentially safe and well-tolerated supportive intervention that could potentially improve chemotherapy tolerability. However, given the study design and limited sample size, these observations should be interpreted as exploratory and hypothesis-generating rather than confirmatory.

## Supplementary Information

Below is the link to the electronic supplementary material.

Supplementary Material 1

Supplementary Material 2

Supplementary Material 3

## Data Availability

The datasets generated and/or analyzed during the current study are available from the corresponding author on reasonable request.

## Abbreviations

AC: Doxorubicin and Cyclophosphamide
AC-T: Doxorubicin, Cyclophosphamide, and Paclitaxel
ACEIs: Angiotensin Converting Enzyme Inhibitors
AEs: Adverse Events
ANC: Absolute Neutrophil Count
ANOVA: Analysis of Variance
ARBs: Angiotensin Receptor Blockers
ASCO: American Society of Clinical Oncology
ATP: Adenosine Triphosphate
BMI: Body Mass Index
CCBs: Calcium Channel Blockers
CBC: Complete Blood Count
CI: Confidence Interval
CoQ10: Coenzyme Q10
CTCAE: Common Terminology Criteria for Adverse Events
ECOG-PS: Eastern Cooperative Oncology Group Performance Status
eGFR: Estimated Glomerular Filtration Rate
IARC: International Agency for Research on Cancer
IRB: Institutional Review Board
IUD: Intrauterine Device
MDA: Malondialdehyde
ME/CFS: Myalgic Encephalomyelitis/Chronic Fatigue Syndrome
NADH: Nicotinamide Adenine Dinucleotide (Reduced form)
NCI: National Cancer Institute
NSAIDs: Non-Steroidal Anti-Inflammatory Drugs
OR: Odds Ratio
PIPN: Paclitaxel-Induced Peripheral Neuropathy
QoL: Quality of Life
SD: Standard Deviation
TNF-α: Tumor Necrosis Factor alpha
WHO: World Health Organization
