# Supplementary material for: Coenzyme Q10 as an adjunctive strategy to reduce paclitaxel-induced toxicities in breast cancer: a randomized controlled trial
Source: BMC Pharmacol Toxicol. 2026 Jul 4;27:95. doi: 10.1186/s40360-026-01163-7 (PMC13339401; doi:10.1186/s40360-026-01163-7)
Supplement: Supplementary file 1 — Supplementary Material 1 [file 40360_2026_1163_MOESM1_ESM.docx]

| **Adverse events assessment form according to the National Cancer Institute Common Terminology Criteria for Adverse Events (NCI-CTCAE) version 5.0** (1) | | | | | | |
| --- | --- | --- | --- | --- | --- | --- |
| **Patient name:** | |  | | | | |
| **Patient ID:** | |  | | | | |
| **Clinician:** | |  | | | | |
| **Date:** | |  | | | | |
| **Cycle number:** | | **Cycle 1** | **Cycle 2** | **Cycle 3** | **Cycle 4** | |
|  |  | **Tw 1** | **Tw 4** | **Tw 7** | **Tw 10** | |
|  |  | **Tw 2** | **Tw 5** | **Tw 8** | **Tw 11** | |
|  |  | **Tw 3** | **Tw 6** | **Tw 9** | **Tw 12** | |
| **CTCAE v5.0 SOC** | **CTCAE v5.0 Term** | **Grade 1** | **Grade 2** | **Grade 3** | **Grade 4** | **Grade 5** |
| **General disorders and administration site conditions** | **Fatigue** | Fatigue relieved by rest | Fatigue not relieved by rest; limiting instrumental ADL | Fatigue not relieved by rest, limiting self-care ADL | - | - |
|  | A disorder characterized by a state of generalized weakness with a pronounced inability to summon sufficient energy to accomplish daily activities. | | | | | |
| **Nervous system disorders** | **Headache** | Mild pain | Moderate pain; limiting instrumental ADL | Severe pain; limiting self-care ADL | - | - |
|  | A disorder characterized by a sensation of marked discomfort in various parts of the head, not confined to the area of distribution of any nerve. | | | | | |
|  | **Insomnia** | Mild difficulty falling asleep, staying asleep or waking up early | Moderate difficulty falling asleep, staying asleep or waking up early | Severe difficulty in falling asleep, staying asleep or waking up early | - | - |
|  | A disorder characterized by difficulty in falling asleep and/or remaining asleep. | | | | | |
|  | **Peripheral sensory neuropathy** | Asymptomatic | Moderate symptoms; limiting instrumental ADL | Severe symptoms; limiting self-care ADL | Life-threatening consequences: urgent intervention indicated |  |
|  | A disorder characterized by damage or dysfunction of the peripheral sensory nerves. | | | | | |
| **Musculoskeletal and connective tissue disorders** | **Arthralgia** | Mild pain | Moderate pain; limiting instrumental ADL | Severe pain; limiting self-care ADL | - | - |
|  | A disorder characterized by a sensation of marked discomfort in a joint. | | | | | |
|  | **Myalgia** | Mild pain | Moderate pain; limiting instrumental ADL | Severe pain; limiting self-care ADL | - | - |
|  | A disorder characterized by marked discomfort sensation originating from a muscle or group of muscles. | | | | | |
| **Gastrointestinal disorders** | **Nausea** | Loss of appetite without alteration in eating habits | Oral intake decreased without significant weight loss, dehydration or malnutrition | Inadequate oral caloric or fluid intake; tube feeding, TPN, or hospitalization indicated | - | - |
|  | A disorder characterized by a queasy sensation and/or the urge to vomit. | | | | | |
|  | **Vomiting** | Intervention not indicated | Outpatient IV hydration; medical intervention indicated | Tube feeding, TPN, or hospitalization indicated | Life-threatening consequences | Death |
|  | A disorder characterized by the reflexive act of ejecting the contents of the stomach through the mouth. | | | | | |
|  | **Diarrhea** | Increase of <4 stools per day over baseline; mild increase in ostomy output compared to baseline | Increase of 4 - 6 stools per day over baseline; moderate increase in ostomy output compared to baseline | Increase of >=7 stools per day over baseline; incontinence; hospitalization indicated; severe increase in ostomy output compared to baseline; limiting self-care ADL | Life-threatening consequences: urgent intervention indicated | Death |
|  | A disorder characterized by frequent and watery bowel movements. | | | | | |
|  | **Constipation** | Occasional or intermittent symptoms; occasional use of stool softeners, laxatives, dietary modification, or enema | Persistent symptoms with regular use of laxatives or enemas; limiting instrumental ADL | Obstipation with manual evacuation indicated, limiting self-care ADL | Life-threatening consequences: urgent intervention indicated | Death |
|  | A disorder characterized by irregular and infrequent or difficult evacuation of the bowels. | | | | | |
|  | **oral Mucositis** | Asymptomatic or mild symptoms; intervention not indicated | Moderate pain; not interfering with oral intake; modified diet indicated | Severe pain; interfering with oral intake | Life-threatening consequences: urgent intervention indicated | Death |
|  | A disorder characterized by inflammation of the oral mucosal. | | | | | |
|  | **Dry mouth** | Symptomatic (e.g., dry or thick saliva) without significant dietary alteration; unstimulated saliva flow >0.2 ml/min | Moderate symptoms; oral intake alterations (e.g., copious water, other lubricants, diet limited to purees and/or soft, moist foods); unstimulated saliva 0.1 to 0.2 ml/min | Inability to adequately aliment orally; tube feeding or TPN indicated; unstimulated saliva <0.1 ml/min | - | - |
|  | A disorder characterized by reduced salivary flow in the oral cavity. | | | | | |
| **Renal and urinary disorders** | **Urinary tract pain** | Mild pain | Moderate pain; limiting instrumental ADL | Severe pain; limiting self-care ADL | - | - |
|  | A disorder characterized by a sensation of marked discomfort in the urinary tract. | | | | | |
| **Blood and lymphatic system disorders** | **Anemia** | Hemoglobin (Hgb) <LLN - 10.0 g/dL; <LLN - 6.2 mmol/L; <LLN - 100 g/L | Hgb <10.0 - 8.0 g/dL; <6.2 - 4.9 mmol/L; <100 - 80g/L | Hgb <8.0 g/dL; <4.9 mmol/L; <80 g/L; transfusion indicated | Life-threatening consequences: urgent intervention indicated | Death |
|  | A disorder characterized by an reduction in the amount of hemoglobin in 100 ml of blood. Signs and symptoms of anemia may include pallor of the skin and mucous membranes, shortness of breath, palpitations of the heart, soft systolic murmurs, lethargy, and fatigability. | | | | | |
|  | **Neutrophil count decreased** | <LLN - 1500/mm3; <LLN - 1.5 x 10e9 /L | <1500 - 1000/mm3; <1.5 - 1.0 x 10e9 /L | <1000 - 500/mm3; <1.0 - 0.5 x 10e9 /L | <500/mm3; <0.5 x 10e9 /L | - |
|  | A finding based on laboratory test results indicate a decrease in number of neutrophils in a blood specimen. | | | | | |
|  | **Platelet count decreased** | <LLN - 75,000/mm3; <LLN - 75.0 x 10e9 /L | <75,000 - 50,000/mm3; <75.0 - 50.0 x 10e9 /L | <50,000 - 25,000/mm3; <50.0 - 25.0 x 10e9 /L | <25,000/mm3; <25.0 x 10e9 /L | - |
|  | A finding based on laboratory test results that indicate a decrease in number of platelets in a blood specimen. | | | | | |
|  | **Febrile neutropenia** | - | - | ANC <1000/mm3 with a single temperature of >38.3 degrees C (101 degrees F) or a sustained temperature of >=38 degrees C (100.4 degrees F) for more than one hour | Life-threatening consequences: urgent intervention indicated | Death |
|  | A disorder characterized by an ANC <1000/mm3 and a single temperature of >38.3 degrees C (101 degrees F) or a sustained temperature of >=38 degrees C (100.4 degrees F) for more than one hour. | | | | | |
| **Skin and subcutaneous tissue disorders** | **Alopecia** | Hair loss of <50% of normal for that individual that is not obvious from a distance but only on close inspection; a different hair style may be required to cover the hair loss but it does not require a wig or hair piece to camouflage | Hair loss of >=50% normal for that individual that is readily apparent to others; a wig or hair piece is necessary if the patient desires to completely camouflage the hair loss; associated with psychosocial impact |  |  |  |
|  | A disorder is characterized by a decrease in density of hair compared to normal for a given individual at a given age and body location. | | | | | |
|  | **Nail changes** | Present | - | - | - | - |
|  | A disorder characterized by a change in the nails. | | | | | |

1. CTEP Trial Development and Conduct - NCI [Internet]. 2025 [cited 2025 Nov 9]. Available from: https://dctd.cancer.gov/research/ctep-trials/trial-development
