# Supplementary material for: Coenzyme Q10 as an adjunctive strategy to reduce paclitaxel-induced toxicities in breast cancer: a randomized controlled trial
Source: BMC Pharmacol Toxicol. 2026 Jul 4;27:95. doi: 10.1186/s40360-026-01163-7 (PMC13339401; doi:10.1186/s40360-026-01163-7)
Supplement: Supplementary file 3 — Supplementary Material 3 [file 40360_2026_1163_MOESM3_ESM.docx]

**Supplementary Table 1: Number of patients experienced each event at Grade 1-4, based on their worst (maximum) grade during the treatment period. Grading is according to the National Cancer Institute Common Terminology Criteria for Adverse Events version 5.0**

|  |  | Control arm (n=26) | | | |  | CoQ 10 arm n= (25) | | | |
| --- | --- | --- | --- | --- | --- | --- | --- | --- | --- | --- |
|  | No event  n/26 (%) | Grade 1  n/26 (%) | Grade 2  n/26 (%) | Grade 3  n/26 (%) | Grade 4  n/26 (%) | No event  n/25 (%) | Grade 1  n/25 (%) | Grade 2  n/25 (%) | Grade 3  n/25 (%) | Grade 4  n/25 (%) |
| Nervous system disordes |  |  |  |  |  |  |  |  |  |  |
| Peripheral neuropathy | 0 (0.00) | 1 (3.84) | 19 (73.07) | 6 (23.07) | 0 (0.00) | 0 (0.00) | 8 (32.00) | 14 (56.00) | 3 (12.00) | 0 (0.00) |
| General disorders | | | | | | | | | | |
| Fatigue | 0 (0.00) | 2 (7.69) | 9 (34.61) | 15 (57.69) | - | 0 (0.00) | 4 (16.00) | 14 (56.00) | 7 (28.00) | - |
| Other nervous system disordes | | | | | | | | | | |
| Headache | 0 (0.00) | 2 (7.00) | 14 (53.8) | 10 (38.4) | - | 0 (0.00) | 1 (4.00) | 19 (76.00) | 5 (36.00) | - |
| Insomnia | 0 (0.00) | 1 (3.84) | 16 (61.53) | 9 (34.6) | - | 0 (0.00) | 4 (16.00) | 18 (72.00) | 3 (12.00) | - |
| Musculoskeletal disorders | | | | | | | | | | |
| Arthralgia | 0 (0.00) | 1(3.84) | 13 (50.00) | 12 (46.15) | - | 0 (0.00) | 5(20.00) | 13 (52.00) | 7 (28.00) | - |
| Myalgia | 0 (0.00) | 2 (7.69) | 12 (46.15) | 12 (46.15) | - | 0 (0.00) | 6 (24.00) | 10 (40.00) | 9 (36.00) | - |
| Gastrointestinal disorders | | | | | | | | | | |
| Mucositis | 0 (0.00) | 15 (57.69) | 9 (34.61) | 2 (7.69) | 0 (0.00) | 0 (0.00) | 17 (68.00) | 8 (32.00) | 0 (0.00) | 0 (0.00) |
| Diarrhea | 0 (0.00) | 11 (42.30) | 11 (42.30) | 3 (15.38) | 0 (0.00) | 0 (0.00) | 12 (48.00) | 12 (48.00) | 0 (0.00) | 0 (0.00) |
| Dry mouth | 1 (3.84) | 20 (76.92) | 5 (19.23) | 0 (0.00) | - | 1 (4.00) | 18 (72.00) | 6 (24.00) | 0 (0.00) | - |
| Nausea | 0 (0.00) | 16 (64.00) | 9 (34.61) | 1 (3.84) | 0 (0.00) | 0 (0.00) | 12 (48.00) | 13 (52.00) | 0 (0.00) | 0 (0.00) |
| Vomiting | 8 (30.76) | 14 (53.84) | 3 (11.53) | 1 (3.84) | 0 (0.00) | 5 (20.00) | 17 (68.00) | 3 (12.00) | 0 (0.00) | 0 (0.00) |
| constipation | 13 (50.00) | 6 (23.07) | 7 (26.92) | 0 (0.00) | 0 (0.00) | 16 (64.00) | 5 (20.00) | 4 (16.00) | 0 (0.00) | 0 (0.00) |
| Urinary tract pain | 5 (19.23) | 16 (61.53) | 5 (19.23) | 0 (0.00) | - | 3 (12.00) | 19 (76 .00) | 4 (16.00) | 0 (0.00) | - |
| Hematological disorders | | | | | | | | | | |
| Anemia | 0 (0.00) | 17 (65.38) | 6 (23.07) | 3 (11.53) | 0 (0.00) | 2 (8.00) | 18 (72.00) | 4 (16.00) | 1(4.00) | 0 (0.00) |
| Neutropenia | 16 (61.53) | 6 (23.07) | 1 (3.84) | 3 (11.53) | 0 (0.00) | 20 (80.00) | 2 (8.00) | 3 (12.00) | 0 (0.00) | 0 (0.00) |
| Trombocitopenia | 22 (84.61) | 4 (15.38) | 0 (0.00) | 0 (0.00) | 0 (0.00) | 22 (88.00) | 3 (12.00) | 0 (0.00) | 0 (0.00) | 0 (0.00) |
| Febrile neutropenia | 23 (88.64) | - | - | 3 (11.53) | 0 (0.00) | 25 (100.00) | - | - | 0 (0.00) | 0 (0.00) |

Grading was performed according to the National Cancer Institute Common Terminology Criteria for Adverse Events (CTCAE), version 5.0. Data are presented as number of patients (percentage) within each treatment arm (Control: n = 26; CoQ10: n = 25). “No event” indicates patients who did not experience the adverse event at any grade.

**Supplementary Table 2. Summary of primary and secondary outcomes with FDR-corrected significance**

| **Outcome category** | **Specific outcome** | **Week** | **Uncorrected p-value** | **corrected p-value** | **Significant (Yes=1)** |
| --- | --- | --- | --- | --- | --- |
| Neurological / sensory | Neuropathy | 10 | 0.0230 | 0.05 | 1 |
|  | Neuropathy | 11 | 0.0080 | 0.04 | 1 |
|  | Neuropathy | 12 | 0.0010 | 0.02 | 1 |
| Other nervous system disorders | Insomnia | 9 | 0.0030 | 0.03 | 1 |
|  | Insomnia | 10 | 0.0010 | 0.02 | 1 |
|  | Insomnia | 11 | 0.0005 | 0.01 | 1 |
|  | Headache | 10 | 0.0010 | 0.02 | 1 |
|  | Headache | 11 | 0.0005 | 0.01 | 1 |
| General symptoms | Fatigue | 9 | 0.0160 | 0.04 | 1 |
|  | Fatigue | 10 | 0.0090 | 0.04 | 1 |
|  | Fatigue | 11 | 0.0005 | 0.01 | 1 |
| Musculoskeletal | Arthralgia | 11 | 0.0020 | 0.03 | 1 |
|  | Arthralgia | 12 | 0.0010 | 0.02 | 1 |
|  | Myalgia | 11 | 0.0180 | 0.05 | 1 |
|  | Myalgia | 12 | 0.0040 | 0.03 | 1 |
| Gastrointestinal | Diarrhea | 11 | 0.0020 | 0.03 | 1 |
|  | Diarrhea | 12 | 0.0020 | 0.03 | 1 |
|  | Mucositis | 11 | 0.0005 | 0.01 | 1 |
|  | Mucositis | 12 | 0.0010 | 0.02 | 1 |
| Hematological | Hemoglobin | 12 | 0.0090 | 0.04 | 1 |
| Dermatological | Nail changes | 12 | 0.0090 | 0.04 | 1 |
| Cardiac | Ejection fraction | 12 | 0.0050 | 0.03 | 1 |

FDR (False Discovery Rate)-corrected p-values were calculated using the Benjamini-Hochberg procedure to account for multiple comparisons. Significant outcomes (Yes=1) indicate FDR-corrected p ≤ 0.05.

| **Supplementary Table 3: Frequencies of different CTCAE severity grades of peripheral neuropathy in control group versus CoQ10 group experienced during Taxol chemotherapy** | | | | | | | | | | | | | | |
| --- | --- | --- | --- | --- | --- | --- | --- | --- | --- | --- | --- | --- | --- | --- |
|  | ***Control group arm***  ***(n= 26)*** | | | | | | ***Coenzyme Q10 arm***  ***(n= 25)*** | | | | | | ***Odds ratio***  ***[95% C.I.]*** | ***p-value*** |
|  | ***Median [IQR]*** | ***No event (%)*** | ***G1 (%)*** | ***G2 (%)*** | ***G3 (%)*** | ***G4 (%)*** | ***Median [IQR]*** | ***No event (%)*** | ***G1 (%)*** | ***G2 (%)*** | ***G3 (%)*** | ***G4 (%)*** |  |  |
| ***Tw1*** | *1.00  [1.00-2.00]* | *0 (0.00)* | *19(73.08)* | *7(26.92)* | *0 (0.00)* | *0 (0.00)* | *1.00*  *[.00*  *1.00]* | *7 (28)* | *13(52)* | *5(20)* | *0 (0.00)* | *0 (0.00)* |  | *0.052* |
| ***Tw2*** | *1.00  [1.00-2.00]* | *2(7.69)* | *15(57.69)* | *8(30.77)* | *1(3.85)* | *0 (0.00)* | *1.00  [1.00-2.00]* | *0 (0.00)* | *18(72)* | *7(28)* | *0 (0.00)* | *0 (0.00)* |  | *0.874* |
| ***Tw3*** | *2.00 [2.00-2.00]* | *0 (0.00)* | *11(42.31)* | *15(57.69)* | *0 (0.00)* | *0 (0.00)* | *1.00  [1.00-2.00]* | *0 (0.00)* | *17(68)* | *8(32)* | *0 (0.00)* | *0 (0.00)* |  | *0.069* |
| ***Tw4*** | *2.00 [2.00-2.00]* | *0 (0.00)* | *5(19.23)* | *19(73.08)* | *2(7.69)* | *0 (0.00)* | *2.00 [2.00-2.00]* | *0 (0.00)* | *9(36)* | *16(64)* | *0 (0.00)* | *0 (0.00)* |  | *0.103* |
| ***Tw5*** | *2.00 [2.00-2.00]* | *0 (0.00)* | *7(26.92)* | *18(69.23)* | *1(3.85)* | *0 (0.00)* | *2.00 [2.00-2.00]* | *0 (0.00)* | *9(36)* | *15(60)* | *1(4)* | *0 (0.00)* |  | *0.524* |
| ***Tw6*** | *2.00 [2.00-2.00]* | *0 (0.00)* | *2(7.69)* | *23(88.46)* | *1(3.85)* | *0 (0.00)* | *2.00 [2.00-2.00]* | *0 (0.00)* | *8(32)* | *14(56)* | *3(12)* | *0 (0.00)* |  | *0.213* |
| ***Tw7*** | *2.00 [2.00-2.00]* | *0 (0.00)* | *8(30.77)* | *17(65.38)* | *1(3.85)* | *0 (0.00)* | *2.00 [2.00-2.00]* | *0 (0.00)* | *9(36)* | *16(64)* | *0 (0.00)* | *0 (0.00)* |  | *0.568* |
| ***Tw8*** | *2.00 [2.00-2.00]* | *0 (0.00)* | *2(7.69)* | *22(84.62)* | *2(7.69)* | *0 (0.00)* | *2.00 [2.00-2.00]* | *0 (0.00)* | *8(32)* | *14(56)* | *3(12)* | *0 (0.00)* |  | *0.157* |
| ***Tw9*** | *2.00 [2.00-2.00]* | *0 (0.00)* | *3(11.54)* | *19(73.08)* | *4(15.38)* | *0 (0.00)* | *2.00 [2.00-2.00]* | *0 (0.00)* | *8(32)* | *14(56)* | *3(12)* | *0 (0.00)* |  | *0.141* |
| ***Tw10*** | *2.00 [2.00-2.00]* | *0 (0.00)* | *3(11.54)* | *20(76.92)* | *4(15.38)* | *0 (0.00)* | *2.00 [2.00-2.00]* | *0 (0.00)* | *8(32)* | *17(68)* | *0 (0.00)* | *0 (0.00)* | *0.193 (0.0 47-0.796)* | *0.023* |
| ***Tw11*** | *2.00 [2.00-2.00]* | *0 (0.00)* | *2(7.69)* | *20(76.92)* | *4(15.38)* | *0 (0.00)* | *2.00 [2.00-2.00]* | *0 (0.00)* | *9(36)* | *16(64)* | *0 (0.00)* | *0 (0.00)* | *0.110-(0.022-0.555)* | *0.008* |
| ***Tw12*** | *2.00 [2.00-2.00]* | *1(3.85)* | *4(15.38)* | *20(76.92)* | *1(3.85)* | *0 (0.00)* | *1.00  [1.00-2.00]* | *0 (0.00)* | *18(72)* | *7(28)* | *0 (0.00)* | *0 (0.00)* | *.107 (.030 .384)* | *0.001* |

Frequencies and severity of peripheral neuropathy by week (Tw1-Tw12) in patients receiving paclitaxel, comparing the Control group (n = 26) and CoQ10 group (n = 25). Data show the median [interquartile range], number of patients with “No event” or CTCAE Grades 1-4, odds ratios with 95% confidence intervals, and p-values. Grading was performed according to the National Cancer Institute Common Terminology Criteria for Adverse Events (CTCAE), version 5.0.

**Supplementary Table 4: Patient-Level Kaplan-Meier Estimates of Time to Grade ≥2 PIPN**

| **Drug Group** | **Patient ID** | **Time (weeks)** | **Status (1=event, 0=censored)** | **Cumulative Proportion Surviving** | **Std. Error** | **N of Cumulative Events** | **N Remaining** |
| --- | --- | --- | --- | --- | --- | --- | --- |
| 0 | 1 | 6 | 1 | 0.962 | 0.038 | 1 | 25 |
| 0 | 3 | 9 | 1 | - | - | 2 | 24 |
| 0 | 23 | 9 | 1 | - | - | 3 | 23 |
| 0 | 26 | 9 | 1 | 0.846 | 0.071 | 4 | 22 |
| 0 | 6 | 11 | 1 | 0.808 | 0.077 | 5 | 21 |
| 0 | 9 | 12 | 1 | - | - | 6 | 20 |
| 0 | 13 | 12 | 1 | 0.731 | 0.087 | 7 | 19 |
| 0 | 5 | 13 | 1 | - | - | 8 | 18 |
| 0 | 10 | 13 | 1 | 0.654 | 0.093 | 9 | 17 |
| 0 | 4 | 16 | 1 | 0.615 | 0.095 | 10 | 16 |
| 0 | 16 | 17 | 1 | 0.577 | 0.097 | 11 | 15 |
| 0 | 18 | 19 | 1 | 0.538 | 0.098 | 12 | 14 |
| 0 | 20 | 20 | 1 | - | - | 13 | 13 |
| 0 | 24 | 20 | 1 | - | - | 14 | 12 |
| 0 | 25 | 20 | 1 | 0.423 | 0.097 | 15 | 11 |
| 0 | 19 | 21 | 1 | 0.385 | 0.095 | 16 | 10 |
| 0 | 12 | 28 | 1 | - | - | 17 | 9 |
| 0 | 22 | 28 | 1 | 0.308 | 0.091 | 18 | 8 |
| 0 | 8 | 29 | 1 | - | - | 19 | 7 |
| 0 | 17 | 29 | 1 | - | - | 20 | 6 |
| 0 | 21 | 29 | 1 | 0.192 | 0.077 | 21 | 5 |
| 0 | 14 | 32 | 1 | 0.154 | 0.071 | 22 | 4 |
| 0 | 7 | 45 | 1 | 0.115 | 0.063 | 23 | 3 |
| 0 | 15 | 55 | 1 | 0.077 | 0.052 | 24 | 2 |
| 0 | 2 | 80 | 1 | 0.038 | 0.038 | 25 | 1 |
| 0 | 11 | 89 | 1 | 0.000 | 0.000 | 26 | 0 |
| 1 | 1 | 9 | 1 | 0.960 | 0.039 | 1 | 24 |
| 1 | 11 | 10 | 1 | 0.920 | 0.054 | 2 | 23 |
| 1 | 19 | 11 | 1 | 0.880 | 0.065 | 3 | 22 |
| 1 | 2 | 12 | 1 | 0.840 | 0.073 | 4 | 21 |
| 1 | 13 | 16 | 1 | - | - | 5 | 20 |
| 1 | 14 | 16 | 1 | 0.760 | 0.085 | 6 | 19 |
| 1 | 20 | 20 | 1 | 0.720 | 0.090 | 7 | 18 |
| 1 | 18 | 24 | 1 | 0.680 | 0.093 | 8 | 17 |
| 1 | 21 | 25 | 1 | 0.640 | 0.096 | 9 | 16 |
| 1 | 22 | 27 | 1 | - | - | 10 | 15 |
| 1 | 25 | 27 | 1 | 0.560 | 0.099 | 11 | 14 |
| 1 | 3 | 28 | 1 | 0.520 | 0.100 | 12 | 13 |
| 1 | 9 | 30 | 1 | 0.480 | 0.100 | 13 | 12 |
| 1 | 8 | 34 | 1 | 0.440 | 0.099 | 14 | 11 |
| 1 | 12 | 35 | 1 | 0.400 | 0.098 | 15 | 10 |
| 1 | 17 | 37 | 1 | 0.360 | 0.096 | 16 | 9 |
| 1 | 4 | 44 | 1 | 0.320 | 0.093 | 17 | 8 |
| 1 | 23 | 77 | 0 | - | - | 17 | 7 |
| 1 | 24 | 83 | 0 | - | - | 17 | 6 |
| 1 | 10 | 93 | 0 | - | - | 17 | 5 |
| 1 | 7 | 94 | 0 | - | - | 17 | 4 |
| 1 | 6 | 96 | 0 | - | - | 17 | 3 |
| 1 | 16 | 96 | 0 | - | - | 17 | 2 |
| 1 | 1 | 97 | 0 | - | - | 17 | 1 |
| 1 | 5 | 97 | 0 | - | - | 17 | 0 |

Data are shown separately for the Control group (0) and CoQ10 group (1). Columns indicate Patient ID, time to event in weeks, event status (1 = PIPN Grade ≥2 occurred; 0 = censored), cumulative proportion surviving without Grade ≥2 PIPN, standard error, number of cumulative events, and number of patients remaining at risk at each time point. Survival estimates were calculated using the Kaplan-Meier method, and grading follows the NCI CTCAE version 5.0.

**Supplementary Table 5: Frequencies of different CTCAE severity grades of Insomnia in control group versus CoQ10 group experienced during Taxol chemotherapy**

|  | ***Control group group***  ***(n= 26)*** | | | | | | ***Coenzyme Q10 group***  ***(n= 25)*** | | | | | | ***Odds ratio***  ***[95% C.I.]*** | ***p-value*** |
| --- | --- | --- | --- | --- | --- | --- | --- | --- | --- | --- | --- | --- | --- | --- |
|  | ***Median [IQR]*** | ***No event (%)*** | ***G1 (%)*** | ***G2 (%)*** | ***G3 (%)*** | ***G4 (%)*** | ***Median [IQR]*** | ***No event (%)*** | ***G1 (%)*** | ***G2 (%)*** | ***G3 (%)*** | ***G4 (%)*** |  |  |
| Tw1 | *1.00 [.00-1.00]* | *7(28)* | *13(50)* | *5(19.23)* | *0 (0.00)* | *0 (0.00)* | *1.00 [1.00-1.50]* | *3(12)* | *16(64)* | *6(24)* | *0 (0.00)* | *0 (0.00)* |  | *0.203* |
| Tw2 | *1.00 [.00-2.00]* | *5(20)* | *10(38.46)* | *11(42.31)* | *0 (0.00)* | *0 (0.00)* | *1.00 [.00-2.00]* | *9(36)* | *8(32)* | *8(32)* | *0 (0.00)* | *0 (0.00)* |  | *0.237* |
| Tw3 | *1.00 [1.00-1.25]* | *4(16)* | *16(61.54)* | *6(23.08)* | *0 (0.00)* | *0 (0.00)* | *1.00 [.00-2.00]* | *10(40)* | *8(32)* | *7(28)* | *0 (0.00)* | *0 (0.00)* |  | *0.322* |
| Tw4 | *1.00 [1.00-2.00]* | *2(8)* | *17(65.38)* | *7(26.92)* | *0 (0.00)* | *0 (0.00)* | *1.00 [1.00-1.50]* | *1(4)* | *18(72)* | *6(24)* | *0 (0.00)* | *0 (0.00)* |  | *1.000* |
| Tw5 | *1.50 1[.00-2.00]* | *3(12)* | *10(38.46)* | *13(50)* | *0 (0.00)* | *0 (0.00)* | *1.00 [1.00-2.00]* | *4(16)* | *11(44)* | *10(40)* | *0 (0.00)* | *0 (0.00)* |  | *0.454* |
| Tw6 | *1.00 [.00-2.00]* | *1(4)* | *13(50)* | *11(42.31)* | *1(3.85)* | *0 (0.00)* | *1.00 [.00-2.00]* | *7(28)* | *11(44)* | *7(28)* | *0 (0.00)* | *0 (0.00)* |  | *0.329* |
| Tw7 | *1.00 [1.00-2.00]* | *1(4)* | *13(50)* | *11(42.31)* | *1(3.85)* | *0 (0.00)* | *1.00 [1.00-2.00]* | *0 (0.00)* | *18(72)* | *7(28)* | *0 (0.00)* | *0 (0.00)* |  | *0.236* |
| Tw8 | *1.00 [1.00-2.00]* | *0 (0.00)* | *15(57.69)* | *11(42.31)* | *0 (0.00)* | *0 (0.00)* | *1.00 [1.00-2.00]* | *3(12)* | *15(60)* | *7(28)* | *0 (0.00)* | *0 (0.00)* |  | *0.133* |
| Tw9 | *2.00 [1.00-2.00]* | *0 (0.00)* | *11(42.31)* | *13(50)* | *2(7.69)* | *0 (0.00)* | *1.00 [.00-2.00]* | *8(32)* | *10(40)* | *7(28)* | *0 (0.00)* | *0 (0.00)* | *0.180(0.058-0.561)* | *0.003* |
| Tw10 | *2.00 [1.00-2.00]* | *6(24)* | *7(26.92)* | *14(53.85)* | *3(11.54)* | *0 (0.00)* | *1.00 [.00-2.00]* | *9(36)* | *9(36)* | *6(24)* | *1(4)* | *0 (0.00)* | *0.149(0.047-0.471)* | *0.001* |
| Tw11 | *2.00 [2.00-2.25]* | *0 (0.00)* | *4(15.38)* | *16(61.54)* | *5(19.23)* | *0 (0.00)* | *.00 [.00-2.00]* | *13(52)* | *8(32)* | *1(4)* | *3(12)* | *0 (0.00)* | *0.048(0.012-0.190)* | *0.000* |
| Tw12 | *2.00 [2.00-2.25]* | *0 (0.00)* | *5(19.23)* | *15(57.69)* | *5(19.23)* | *0 (0.00)* | *.00 [.00-2.00]* | *13(52)* | *9(36)* | *1(4)* | *2(8)* | *0 (0.00)* | *0.034(0.008-0.150)* | *0.000* |

Data are shown weekly (Tw1-Tw12) for the Control group (n = 26) and CoQ10 group (n = 25). Columns include median [IQR], number and percentage of patients with no event, Grade 1-4 insomnia, odds ratio [95% CI], and p-value comparing the two groups. Grading was performed according to NCI CTCAE version 5.0.

**Supplementary Table 6: Frequencies of different CTCAE severity grades of headache in control group versus CoQ10 group experienced during Taxol chemotherapy**

|  | ***Control group group***  ***(n= 26)*** | | | | | | ***Coenzyme Q10 group***  ***(n= 25)*** | | | | | | ***Odds ratio***  ***[95% C.I.]*** | ***p-value*** |
| --- | --- | --- | --- | --- | --- | --- | --- | --- | --- | --- | --- | --- | --- | --- |
|  | ***Median [IQR]*** | ***No event (%)*** | ***G1 (%)*** | ***G2 (%)*** | ***G3 (%)*** | ***G4 (%)*** | ***Median [IQR]*** | ***No event (%)*** | ***G1 (%)*** | ***G2 (%)*** | ***G3 (%)*** | ***G4 (%)*** |  |  |
| *Tw1* | *1.00 [.00-1.00]* | *11(42.31)* | *11(42.31)* | *3(11.54)* | *0 (0.00)* | *0 (0.00)* | *1.00 [.00-1.00]* | *12(48)* | *11(44)* | *2(8)* | *0 (0.00)* | *0 (0.00)* |  | *0.809* |
| *Tw2* | *1.00 [1.00-2.00]* | *3(11.54)* | *12(46.15)* | *11(42.31)* | *0 (0.00)* | *0 (0.00)* | *1.00 [1.00-2.00]* | *1(4)* | *12(48)* | *10(40)* | *2(8)* | *0 (0.00)* |  | *0.386* |
| *Tw3* | *2.00 [1.00-2.00]* | *2(7.69)* | *9(34.62)* | *14(53.85)* | *0 (0.00)* | *0 (0.00)* | *2.00 [1.00-2.00]* | *0 (0.00)* | *9(36)* | *15(60)* | *1(4)* | *0 (0.00)* |  | *0249* |
| *Tw4* | *2.00 [1.00-2.00]* | *2(7.69)* | *10(38.46)* | *14(53.85)* | *0 (0.00)* | *0 (0.00)* | *2.00 [1.00-2.00]* | *0 (0.00)* | *9(36)* | *16(64)* | *0 (0.00)* | *0 (0.00)* |  | *0.356* |
| ***Tw5*** | *1.00 [1.00-2.00]* | *0 (0.00)* | *14(53.85)* | *12(46.15)* | *0 (0.00)* | *0 (0.00)* | *1.00 [1.00-2.00]* | *0 (0.00)* | *13(52)* | *12(48)* | *0 (0.00)* | *0 (0.00)* |  | *.0895* |
| ***Tw6*** | *2.00 [1.00-2.00]* | *0 (0.00)* | *11(42.31)* | *14(53.85)* | *1(3.85)* | *0 (0.00)* | *2.00 [1.00-2.00]* | *0 (0.00)* | *10(40)* | *14(56)* | *1(4)* | *0 (0.00)* |  | *0.870* |
| ***Tw7*** | *2.00 [1.00-2.00]* | *0 (0.00)* | *12(46.15)* | *13(50)* | *1(3.85)* | *0 (0.00)* | *2.00 [1.00-2.00]* | *0 (0.00)* | *10(40)* | *15(60)* | *0 (0.00)* | *0 (0.00)* |  | *0.784* |
| *Tw8* | *1.00 [1.00-2.00]* | *0 (0.00)* | *14(53.85)* | *11(42.31)* | *1(3.85)* | *0 (0.00)* | *2.00 [1.00-2.00]* | *0 (0.00)* | *9(36)* | *13(52)* | *3(12)* | *0 (0.00)* |  | *0.151* |
| *Tw9* | *1.00 [1.00-2.00]* | *0 (0.00)* | *11(42.31)* | *12(46.15)* | *3(11.54)* | *0 (0.00)* | *1.00 [1.00-2.00]* | *0 (0.00)* | *16(64)* | *9(36)* | *0 (0.00)* | *0 (0.00)* |  | *0.073* |
| *Tw10* | *2.00 [1.00-2.00]* | *0 (0.00)* | *9(34.62)* | *12(46.15)* | *5(19.23)* | *0 (0.00)* | *1.00 [1.00-1.00]* | *0 (0.00)* | *20(80)* | *5(20)* | *0 (0.00)* | *0 (0.00)* | *0.119(0.034-0.422)* | *0.001* |
| *Tw11* | *2.00 [1.00-3.00]* | *1(3.85)* | *10(38.46)* | *5(19.23)* | *9(34.62)* | *0 (0.00)* | *1.00 [.00-1.00]* | *10(40)* | *12(48)* | *3(12)* | *0 (0.00)* | *0 (0.00)* | *0.106(0.032-0.358)* | *0.000* |
| *Tw12* | *2.00 [1.00-3.00]* | *1(3.85)* | *10(38.46)* | *6(23.08)* | *8(30.77)* | *0 (0.00)* | *1.00 [.00-1.00]* | *10(40)* | *13(52)* | *2(8)* | *0 (0.00)* | *0 (0.00)* | *0.087(0.024-.0317)* | *0.000* |

Data are shown weekly (Tw1-Tw12) for the Control group (n = 26) and CoQ10 group (n = 25). Columns include median [IQR], number and percentage of patients with no event, Grade 1-4 headache, odds ratio [95% CI], and p-value comparing the two groups. Grading was performed according to NCI CTCAE version 5.0.

**Supplementary Table 7: Frequencies of different CTCAE severity grades of fatigue in control group versus CoQ10 group experienced during Taxol chemotherapy**

|  | ***Control group group***  ***(n= 26)*** | | | | | | ***Coenzyme Q10 group***  ***(n= 25)*** | | | | | | ***Odds ratio***  ***[95% C.I.]*** | ***p-value*** |
| --- | --- | --- | --- | --- | --- | --- | --- | --- | --- | --- | --- | --- | --- | --- |
|  | ***Median [IQR]*** | ***No event (%)*** | *G1 (%)* | *G2 (%)* | *G3 (%)* | *G4 (%)* | *Median [IQR]* | *No event (%)* | *G1 (%)* | *G2 (%)* | *G3 (%)* | *G4 (%)* |  |  |
| *Tw1* | *1..00[1.00-1.25]* | *3 ( 11.54)* | *3( 11.54)* | *3( 11.54)* | *3(11.54)* | *0 (0.00)* | *1.00 [.00-2.00]* | *8 ( 32)* | *10( 40)* | *7( 28)* | *0 (0.00)* | *0 (0.00)* |  | *0.323* |
| *Tw2* | *2.00 [1.00-2.00]* | *2 ( 7.69)* | *5( 19.23)* | *18( 69.23)* | *1(3.85)* | *0 (0.00)* | *1.00 [1.00-2.00]* | *4 ( 16)* | *3( 12)* | *17( 68)* | *1(4)* | *0 (0.00)* |  | *0.816* |
| *Tw3* | *2.00 [1.75-2.00]* | *1 ( 3.85)* | *5( 11.54)* | *18( 69.23)* | *2(7.69)* | *0 (0.00)* | *2.00 21.00-2.00]* | *0 (0.00)* | *5( 20)* | *19( 76)* | *1(4)* | *0 (0.00)* |  | *0.951* |
| *Tw4* | *2.00 [1.75-2.00]* | *1 ( 3.85)* | *5( 15.38)* | *17( 65.38)* | *3(11.54)* | *0 (0.00)* | *2.00 [1.50-2.00]* | *0 (0.00)* | *6( 24)* | *17( 68)* | *2(8)* | *0 (0.00)* |  | *0.846* |
| *Tw5* | *2.00[1.00-2.00]* | *0 (0.00)* | *8( 30.77)* | *15( 57.69)* | *3(11.54)* | *0 (0.00)* | *2.00 [2.00-2.00]* | *0 (0.00)* | *5( 20)* | *15( 60)* | *5(20)* | *0 (0.00)* |  | *0.282* |
| *Tw6* | *2.00[1.75-2.00]* | *0 (0.00)* | *6( 15.38)* | *17( 65.38)* | *3(11.54)* | *0 (0.00)* | *2.00 [2.00-2.00]* | *0 (0.00)* | *5( 20)* | *18( 72)* | *2(8)* | *0 (0.00)* |  | *1.000* |
| *Tw7* | *2.00 [2.00-2.00]* | *0 (0.00)* | *5( 11.54)* | *16( 61.54)* | *5(19.23)* | *0 (0.00)* | *2.00 [2.00-2.00]* | *0 (0.00)* | *5( 20)* | *18( 72)* | *2(8)* | *0 (0.00)* |  | *0.473* |
| *Tw8* | *2.00[1.75-2.00]* | *0 (0.00)* | *6( 11.54)* | *15( 57.69)* | *5(19.23)* | *0 (0.00)* | *2.00 [1.50-2.00]* | *0 (0.00)* | *6( 24)* | *17( 68)* | *2(8)* | *0 (0.00)* |  | *0.500* |
| *Tw9* | *2.00[2.00-3.00]* | *0 (0.00)* | *4( 11.54)* | *11( 42.31)* | *11(42.31)* | *0 (0.00)* | *2.00 [2.00-2.00]* | *0 (0.00)* | *5( 20)* | *19( 76)* | *1(4)* | *0 (0.00)* | *0.227(0.068-0.761)* | *0.016* |
| *Tw10* | *2.50 [2.00-3.00]* | *0 (0.00)* | *5( 11.54)* | *8( 30.77)* | *13(50)* | *0 (0.00)* | *2.00 [1.50-2.00]* | *0 (0.00)* | *6( 24)* | *18( 72)* | *1(4)* | *0 (0.00)* | *0.211(0.066-0.673)* | *0.009* |
| *Tw11* | *2.00 [2.00-3.00]* | *0 (0.00)* | *3( 11.54)* | *12( 46.15)* | *11(42.31)* | *0 (0.00)* | *1.00 [1.00-2.00]* | *0 (0.00)* | *17( 68)* | *8( 32)* | *0 (0.00)* | *0 (0.00)* | *0.027(0.005-0.143)* | *0.000* |
| *Tw12* | *2.00 [2.00-3.00]* | *0 (0.00)* | *3( 11.54)* | *12( 46.15)* | *11(42.31)* | *0 (0.00)* | *1.00 [1.00-2.00]* | *0 (0.00)* | *18( 72)* | *7( 28)* | *0 (0.00)* | *0 (0.00)* | *0.024(.004-0.127)* | *0.000* |

Weekly data (Tw1-Tw12) are reported for the Control group (n = 26) and CoQ10 group (n = 25). Columns include median [IQR], number and percentage of patients with no event, Grade 1-4 fatigue, odds ratio [95% CI], and p-value comparing the two groups. Grading follows NCI CTCAE version 5.0.

**Supplementary Table 8: Frequencies of different CTCAE severity grades of Arthralgia in control group versus CoQ10 group experienced during Taxol chemotherapy**

|  | ***Control group group***  ***(n= 26)*** | | | | | | ***Coenzyme Q10 group***  ***(n= 25)*** | | | | | | *Odds ratio*  *[95% C.I.]* | ***p-value*** |
| --- | --- | --- | --- | --- | --- | --- | --- | --- | --- | --- | --- | --- | --- | --- |
|  | ***Median [IQR]*** | ***No event (%)*** | ***G1 (%)*** | ***G2 (%)*** | ***G3 (%)*** | ***G4 (%)*** | ***Median [IQR]*** | ***No event (%)*** | ***G1 (%)*** | ***G2 (%)*** | ***G3 (%)*** | ***G4 (%)*** |  |  |
| ***Tw1*** | *.00 [.00-.00]* | *18 (69.23)* | *5 (19.23)* | *3(11.54)* | *0 ( 0 )* | *0 ( 0 )* | *.00 [.00-.00]* | *21(84)* | *3 (12)* | *1(4)* | *0 ( 0 )* | *0 ( 0 )* |  | *0.613* |
| ***Tw2*** | *.00 [.00-1.00]* | *6 (24)* | *14 (53.85)* | *5(19.23)* | *1 ( 3.85 )* | *0 ( 0 )* | *.00[.00-.00]* | *9 (36)* | *13 (52)* | *3(12)* | *0 ( 0 )* | *0 ( 0 )* |  | *0.206* |
| ***Tw3*** | *1.00 [.75-1.25]* | *7 (26.92)* | *13 (50)* | *5(19.23)* | *1 ( 3.85 )* | *0 ( 0 )* | *1.00 [1.00-2.00]* | *3 (12)* | *15 (60)* | *7(28)* | *0 ( 0 )* | *0 ( 0 )* |  | *0.199* |
| ***Tw4*** | *1.00 [.00-1.25]* | *0 (0.00)* | *10 (38.46)* | *15(57.69)* | *1 ( 3.85 )* | *0 ( 0 )* | *1.00 [1.00-2.00]* | *0 (0.00)* | *15 (60)* | *10(40)* | *0 ( 0 )* | *0 ( 0 )* |  | *0.338* |
| ***Tw5*** | *2.00 [1.00-2.00]* | *0 (0.00)* | *4 (15.38)* | *22(84.62)* | *0 ( 0 )* | *0 ( 0 )* | *1.00 [1.00-2.00]* | *0 (0.00)* | *7 (28)* | *16(64)* | *2 ( 8 )* | *0 ( 0 )* |  | *0.634* |
| ***Tw6*** | *2.00 [2.00-2.00]* | *0 (0.00)* | *5 (19.23)* | *20(76.92)* | *1 ( 3.85 )* | *0 ( 0 )* | *.00 [1.50-2.00]* | *1 (4)* | *5 (20)* | *17(68)* | *2 ( 8 )* | *0 ( 0 )* |  | *0.874* |
| ***Tw7*** | *2.00 [2.00-2.00]* | *0 (0.00)* | *5 (19.23)* | *18(69.23)* | *3 ( 11.54 )* | *0 ( 0 )* | *.00 [1.50-2.00]* | *2 (8)* | *4 (16)* | *15(60)* | *4 ( 16 )* | *0 ( 0 )* |  | *0.875* |
| ***Tw8*** | *2.00 [2.00-2.00]* | *0 (0.00)* | *4 (15.38)* | *20(76.92)* | *2 ( 7.69 )* | *0 ( 0 )* | *.00[2.00-2.00]* | *4 (16)* | *1 (4)* | *19(76)* | *1 ( 4 )* | *0 ( 0 )* |  | *0.414* |
| ***Tw9*** | *2.00 [2.00-2.00]* | *0 (0.00)* | *3 (11.54)* | *20(76.92)* | *3 ( 11.54 )* | *0 ( 0 )* | *.00 [2.00-2.00]* | *0 (0.00)* | *5 (20)* | *15(60)* | *5 ( 20 )* | *0 ( 0 )* |  | *1.000* |
| ***Tw10*** | *2.00 [2.00-2.00]* | *0 (0.00)* | *3 (11.54)* | *18(69.23)* | *5 ( 19.23 )* | *0 ( 0 )* | *.00 [2.00-2.00]* | *0 (0.00)* | *5 (20)* | *15(60)* | *5 ( 20 )* | *0 ( 0 )* |  | *0.653* |
| ***Tw11*** | *2.00 [2.00-3.00]* | *0 (0.00)* | *1 (3.85)* | *18(69.23)* | *7 ( 26.92 )* | *0 ( 0 )* | *.00 [1.00-2.00]* | *0 (0.00)* | *11 (44)* | *12(48)* | *2 ( 8 )* | *0 ( 0 )* | *0.109 (0.027-0.441)* | *0.002* |
| ***Tw12*** | *2.00 [2.00-3.00]* | *0 (0.00)* | *1 (3.85)* | *17(65.38)* | *8 ( 30.77 )* | *0 ( 0 )* | *.00 [1.00-2.00]* | *0 (0.00)* | *12 (48)* | *12(48)* | *1 ( 4 )* | *0 ( 0 )* | *.0.060 (0.012-0.298)* | *0.001* |

Weekly data (Tw1-Tw12) are reported for the Control group (n = 26) and CoQ10 group (n = 25). Columns include median [IQR], number and percentage of patients with no event, Grade 1-4 arthralgia, odds ratio [95% CI], and p-value comparing the two groups. Grading follows NCI CTCAE version 5.0.

**Supplementary Table 9: Frequencies of different CTCAE severity grades of Myalgia in control group versus CoQ10 group experienced during Taxol chemotherapy**

|  | ***Control group group***  ***(n= 26)*** | | | | | | ***Coenzyme Q10 group***  ***(n= 25)*** | | | | | | ***Odds ratio***  ***[95% C.I.]*** | ***p-value*** |
| --- | --- | --- | --- | --- | --- | --- | --- | --- | --- | --- | --- | --- | --- | --- |
|  | ***Median [IQR]*** | ***No event (%)*** | ***G1 (%)*** | ***G2 (%)*** | ***G3 (%)*** | ***G4 (%)*** | ***Median [IQR]*** | ***No event (%)*** | ***G1 (%)*** | ***G2 (%)*** | ***G3 (%)*** | ***G4 (%)*** |  |  |
| *Tw1* | *0.5 [.00-1.00]* | *15 ( 57.69)* | *8 (30.76)* | *3 (11.53)* | *0 (0.00)* | *0 (0.00)* | *.00 [.00-1.00]* | *17 ( 68)* | *8 (32)* | *0 (0.00)* | *0 (0.00)* | *0 (0.00)* |  | *0.125* |
| *Tw2* | *1.00 [.00-2.00]* | *10 ( 38.46)* | *5 (19.23)* | *10 (38.46)* | *1(3.85)* | *0 (0.00)* | *1.00 [.00-2.00]* | *9 ( 36)* | *7 (28)* | *8 (32)* | *1(4)* | *0 (0.00)* |  | *0.644* |
| *Tw3* | *1.00 [1.00-2.00]* | *2 ( 7.69)* | *13 (50)* | *10 (38.46)* | *1(3.85)* | *0 (0.00)* | *1.00 [1.00-2.00]* | *5 ( 20)* | *10 (40)* | *9 (36)* | *1(4)* | *0 (0.00)* |  | *0.827* |
| *Tw4* | *1.50 [2.00-2.00]* | *0 (0.00)* | *12 (46.15)* | *14 (53.84)* | *1(3.85)* | *0 (0.00)* | *1.00 [1.00-2.00]* | *2 ( 8)* | *14 (56)* | *8 (32)* | *1(4)* | *0 (0.00)* |  | *0.228* |
| *Tw5* | *2.00 [2.00-2.00]* | *0 (0.00)* | *3 (11.54)* | *23 (88.46)* | *1(3.85)* | *0 (0.00)* | *1.00 [1.00-2.00]* | *0 (0.00)* | *9 (36)* | *15 (60)* | *1(4)* | *0 (0.00)* |  | *0.175* |
| *Tw6* | *2.00 [2.00-2.00]* | *0 (0.00)* | *2 (7.69)* | *25 (96.15)* | *0 (0.00)* | *0 (0.00)* | *2.00 [1.00-2.00]* | *0 (0.00)* | *8 (32)* | *15 (60)* | *2(8)* | *0 (0.00)* |  | *0.279* |
| *Tw7* | *2.00 [2.00-2.00]* | *0 (0.00)* | *1 (3.85)* | *24 (92.31)* | *1(3.85)* | *0 (0.00)* | *2.00 [1.50-2.00]* | *0 (0.00)* | *6 (24)* | *15 (60)* | *4(16)* | *0 (0.00)* |  | *0.691* |
| *Tw8* | *2.00 [2.00-2.00]* | *0 (0.00)* | *1 (3.85)* | *24 (92.31)* | *1(3.85)* | *0 (0.00)* | *2.00 [1.50-2.00]* | *0 (0.00)* | *6 (24)* | *16 (64)* | *3(12)* | *0 (0.00)* |  | *0.913* |
| *Tw9* | *2.00 [2.00-2.00]* | *0 (0.00)* | *1 (3.85)* | *20 (76.92)* | *5(19.23)* | *0 (0.00)* | *2.00 [1.00-2.00]* | *0 (0.00)* | *7 (28)* | *15 (60)* | *3(12)* | *0 (0.00)* |  | *0.243* |
| *Tw10* | *2.00 [2.00-3.00]* | *0 (0.00)* | *1 (3.85)* | *18 (69.23)* | *7(26.92)* | *0 (0.00)* | *2.00 [1.00-2.00]* | *0 (0.00)* | *7 (28)* | *15 (60)* | *3(12)* | *0 (0.00)* |  | *0.077* |
| *Tw11* | *2.00 [2.00-3.00]* | *0 (0.00)* | *1 (3.85)* | *18 (69.23)* | *7(26.92)* | *0 (0.00)* | *2.00 [1.00-2.00]* | *0 (0.00)* | *9 (36)* | *13 (52)* | *3(12)* | *0 (0.00)* | *0.235 (0.070-0.783)* | *0.018* |
| *Tw12* | *2.00 [2.00-3.00]* | *0 (0.00)* | *1 (3.85)* | *17 (65.38)* | *8(30.77)* | *0 (0.00)* | *2.00 [1.00-2.00]* | *0 (0.00)* | *10 (40)* | *13 (52)* | *2(8)* | *0 (0.00)* | *0.153 (0.043-0.549)* | *0.004* |

Data are shown as median [IQR] and number (%) of patients for each CTCAE grade (G0-G4). Odds ratios with 95% confidence intervals and p-values indicate the effect of CoQ10 versus control at each treatment week (Tw1-Tw12).

**Supplementary Table 10: Frequencies of different CTCAE severity grades of mucositis in control group versus CoQ10 group experienced during Taxol chemotherapy**

|  | ***Control group group***  ***(n= 25)*** | | | | | | ***Coenzyme Q10 group***  ***(n= 26)*** | | | | | | ***Odds ratio***  ***[95% C.I.]*** | ***p-value*** |
| --- | --- | --- | --- | --- | --- | --- | --- | --- | --- | --- | --- | --- | --- | --- |
|  | ***Median [IQR]*** | ***No event (%)*** | ***G1 (%)*** | ***G2 (%)*** | ***G3 (%)*** | ***G4 (%)*** | ***Median [IQR]*** | ***No event (%)*** | ***G1 (%)*** | ***G2 (%)*** | ***G3 (%)*** | ***G4 (%)*** |  |  |
| *Tw1* | *1.00 [.00-1.00]* | *11(42.31)* | *15(57.69)* | *0 (0.00)* | *0 (0.00)* | *0 (0.00)* | *1.00 [.00-1.00]* | *8(32)* | *15(60)* | *2(8)* | *0 (0.00)* | *0 (0.00)* |  | *0.286* |
| *Tw2* | *.00 [.00-1.00]* | *14(53.85)* | *11(42.31)* | *1(3.85)* | *0 (0.00)* | *0 (0.00)* | *.00 [.00-1.00]* | *16(64)* | *6(24)* | *3(12)* | *0 (0.00)* | *0 (0.00)* |  | *0.670* |
| *Tw3* | *1.00 [.00-1.00]* | *8(30.77)* | *18(69.23)* | *0 (0.00)* | *0 (0.00)* | *0 (0.00)* | *1.00 [.00-1.00]* | *10(40)* | *14(56)* | *1(4)* | *0 (0.00)* | *0 (0.00)* |  | *0.635* |
| *Tw4* | *1.00 [.00-1.00]* | *12(46.15)* | *14(53.85)* | *0 (0.00)* | *0 (0.00)* | *0 (0.00)* | *1.00 [.00-1.00]* | *12(48)* | *12(48)* | *1(4)* | *0 (0.00)* | *0 (0.00)* |  | *0.983* |
| *Tw5* | *.00 [.00-1.00]* | *16(61.54)* | *10(38.46)* | *0 (0.00)* | *0 (0.00)* | *0 (0.00)* | *.00 [.00-1.00]* | *15(60)* | *8(32)* | *2(8)* | *0 (0.00)* | *0 (0.00)* |  | *0.738* |
| *Tw6* | *5.00 [.00-1.00]* | *13(50)* | *13(50)* | *0 (0.00)* | *0 (0.00)* | *0 (0.00)* | *.00 [.00-1.00]* | *14(56)* | *7(28)* | *4(16)* | *0 (0.00)* | *0 (0.00)* |  | *0.889* |
| *Tw7* | *.50 [.00-1.00]* | *13(50)* | *13(50)* | *0 (0.00)* | *0 (0.00)* | *0 (0.00)* | *.00 [.00-1.00]* | *15(60)* | *8(32)* | *2(8)* | *0 (0.00)* | *0 (0.00)* |  | *0.671* |
| *Tw8* | *.00 [.00-1.00]* | *14(53.85)* | *12(46.15)* | *0 (0.00)* | *0 (0.00)* | *0 (0.00)* | *.00 [.00-1.00]* | *13(52)* | *10(40)* | *2(8)* | *0 (0.00)* | *0 (0.00)* |  | *0.696* |
| *Tw9* | *.00 [.00-1.00]* | *15(57.69)* | *11(42.31)* | *0 (0.00)* | *0 (0.00)* | *0 (0.00)* | *.00 [.00-1.00]* | *18(72)* | *7(28)* | *0 (0.00)* | *0 (0.00)* | *0 (0.00)* |  | *0.288* |
| *Tw10* | *.50 [.00-1.00]* | *13(50)* | *12(46.15)* | *1(3.85)* | *0 (0.00)* | *0 (0.00)* | *.00 [.00-1.00]* | *15(60)* | *10(40)* | *0 (0.00)* | *0 (0.00)* | *0 (0.00)* |  | *0.412* |
| *Tw11* | *1.00 [.00-2.00]* | *10(38.46)* | *7(26.92)* | *7(26.92)* | *2(7.69)* | *0 (0.00)* | *.00 [.00-1.00]* | *23(92)* | *2(8)* | *0 (0.00)* | *0 (0.00)* | *0 (0.00)* | *0.050 (0.010-0.257)* | *0.000* |
| *Tw12* | *1.00 [.00-2.00]* | *11(42.31)* | *8(30.77)* | *7(26.92)* | *0 (0.00)* | *0 (0.00)* | *.00 [.00-1.00]* | *22(88)* | *3(12)* | *0 (0.00)* | *0 (0.00)* | *0 (0.00)* | *0.090 (0.022-0.377)* | *0.001* |

Median [IQR] and number (%) of patients are reported per CTCAE grade (G0-G4). Odds ratios and p-values show differences between groups at each treatment week.

**Supplementary Table 11: Frequencies of different CTCAE severity grades of Diarrhea in control group versus CoQ10 group experienced during Taxol chemotherapy**

|  | ***Control group group***  ***(n= 25)*** | | | | | | ***Coenzyme Q10 group***  ***(n= 26)*** | | | | | | ***Odds ratio***  ***[95% C.I.]*** | ***p-value*** |
| --- | --- | --- | --- | --- | --- | --- | --- | --- | --- | --- | --- | --- | --- | --- |
|  | ***Median [IQR]*** | ***No event (%)*** | ***G1 (%)*** | ***G2 (%)*** | ***G3 (%)*** | ***G4 (%)*** | ***Median [IQR]*** | ***No event (%)*** | ***G1 (%)*** | ***G2 (%)*** | ***G3 (%)*** | ***G4 (%)*** |  |  |
| *Tw1* | *1.00 [.00-1.00]* | *9(34.62)* | *17(65.38)* | *0 (0.00)* | *0 (0.00)* | *0 (0.00)* | *1.00 [1.00-1.00]* | *5(20)* | *19(76)* | *1(4)* | *0 (0.00)* | *0 (0.00)* |  | *0.184* |
| *Tw2* | *.50 [.00-1.00]* | *13(50)* | *12(46.15)* | *1(3.85)* | *0 (0.00)* | *0 (0.00)* | *.00 [.00-1.00]* | *13(52)* | *11(44)* | *1(4)* | *0 (0.00)* | *0 (0.00)* |  | *0.897* |
| *Tw3* | *1.00 [.00-1.00]* | *7(26.92)* | *14(53.85)* | *3(11.54)* | *1(3.85)* | *0 (0.00)* | *1.00 [1.00-1.00]* | *5(20)* | *19(76)* | *1(4)* | *0 (0.00)* | *0 (0.00)* |  | *0.879* |
| *Tw4* | *1.00 [.00-1.00]* | *9(34.62)* | *15(57.69)* | *2(7.69)* | *0 (0.00)* | *0 (0.00)* | *1.00 [.00-1.00]* | *8(32)* | *14(56)* | *3(12)* | *0 (0.00)* | *0 (0.00)* |  | *0.714* |
| *Tw5* | *1.00 [.00-1.00]* | *12(46.15)* | *12(46.15)* | *2(7.69)* | *0 (0.00)* | *0 (0.00)* | *1.00 [.00-1.00]* | *7(28)* | *13(52)* | *5(20)* | *0 (0.00)* | *0 (0.00)* |  | *0.114* |
| *Tw6* | *.50 [.00-1.00]* | *13(50)* | *9(34.62)* | *4(15.38)* | *0 (0.00)* | *0 (0.00)* | *1.00 [1.00-1.00]* | *5(20)* | *16(64)* | *4(16)* | *0 (0.00)* | *0 (0.00)* |  | *0.084* |
| *Tw7* | *.50 [.00-1.00]* | *13(50)* | *10(38.46)* | *3(11.54)* | *0 (0.00)* | *0 (0.00)* | *.00 [.00-1.00]* | *13(52)* | *11(44)* | *1(4)* | *0 (0.00)* | *0 (0.00)* |  | *0.701* |
| *Tw8* | *1.00 [.75-1.00]* | *6(23.08)* | *16(61.54)* | *4(15.38)* | *0 (0.00)* | *0 (0.00)* | *1.00 [1.00-1.00]* | *4(16)* | *20(80)* | *1(4)* | *0 (0.00)* | *0 (0.00)* |  | *0.830* |
| *Tw9* | *1.00 [.00-1.00]* | *10(38.46)* | *13(50)* | *3(11.54)* | *0 (0.00)* | *0 (0.00)* | *1.00 [.00-1.00]* | *9(36)* | *12(48)* | *4(16)* | *0 (0.00)* | *0 (0.00)* |  | *0.738* |
| *Tw10* | *1.00 [.00-1.00]* | *10(38.46)* | *13(50)* | *2(7.69)* | *1(3.85)* | *0 (0.00)* | *1.00 [.00-1.00]* | *11(44)* | *11(44)* | *3(12)* | *0 (0.00)* | *0 (0.00)* |  | *0.730* |
| *Tw11* | *1.00 [1.00-1.00]* | *4(15.38)* | *21(80.77)* | *0 (0.00)* | *1(3.85)* | *0 (0.00)* | *1.00 [.00-1.00]* | *16(64)* | *9(36)* | *0 (0.00)* | *0 (0.00)* | *0 (0.00)* | *.099(.026-.379)* | *0.001* |
| *Tw12* | *1.00 [1.00-1.00]* | *4(15.38)* | *22(84.62)* | *0 (0.00)* | *0 (0.00)* | *0 (0.00)* | *1.00 [.00-1.00]* | *15(60)* | *10(40)* | *0 (0.00)* | *0 (0.00)* | *0 (0.00)* | *.121(.032-.459)* | *0.002* |

Values include median [IQR] and number (%) of patients per CTCAE grade (G0-G4). Odds ratios with 95% CIs and p-values indicate group differences weekly.

**Supplementary Table 12: Frequencies of different CTCAE severity grades of nausea in control group versus CoQ10 group experienced during Taxol chemotherapy**

|  | ***Control group group***  ***(n= 25)*** | | | | | | ***Coenzyme Q10 group***  ***(n= 26)*** | | | | | | ***P value*** |
| --- | --- | --- | --- | --- | --- | --- | --- | --- | --- | --- | --- | --- | --- |
|  | ***Median [IQR]*** | ***No event (%)*** | ***G1 (%)*** | ***G2 (%)*** | ***G3 (%)*** | ***G4 (%)*** | ***Median [IQR]*** | ***No event (%)*** | ***G1 (%)*** | ***G2 (%)*** | ***G3 (%)*** | ***G4 (%)*** |  |
| Tw1 | *1.00 [1.00-1.00]* | *2(7.69)* | *24(92.31)* | *0 (0.00)* | *0 (0.00)* | *0 (0.00)* | *1.00 [.00-1.00]* | *9(36)* | *14(56)* | *2(8)* | *0 (0.00)* | *0 (0.00)* | *.095* |
| *Tw2* | *1.00 [1.00-1.00]* | *2(7.69)* | *22(84.62)* | *2(7.69)* | *0 (0.00)* | *0 (0.00)* | *1.00 [.00-1.00]* | *7(28)* | *15(60)* | *3(12)* | *0 (0.00)* | *0 (0.00)* | *0.246* |
| *Tw3* | *1.00 [1.00-1.00]* | *3(11.54)* | *22(84.62)* | *1(3.85)* | *0(1)* | *0 (0.00)* | *1.00 [.00-1.00]* | *7(28)* | *18(72)* | *0 (0.00)* | *0 (0.00)* | *0 (0.00)* | *0.107* |
| *Tw4* | *1.00 [1.00-1.00]* | *2(7.69)* | *20(76.92)* | *4(15.38)* | *0 (0.00)* | *0 (0.00)* | *1.00 [1.00-1.00]* | *1(4)* | *20(80)* | *4(16)* | *0 (0.00)* | *0 (0.00)* | *0.750* |
| *Tw5* | *1.00 [1.00-1.00]* | *1(3.85)* | *21(80.77)* | *4(15.38)* | *0 (0.00)* | *0 (0.00)* | *1.00 [1.00-1.50]* | *3(12)* | *16(64)* | *6(24)* | *0(1)* | *0 (0.00)* | *0.913* |
| *Tw6* | *1.00 [1.00-1.00]* | *2(7.69)* | *21(80.77)* | *3(11.54)* | *0 (0.00)* | *0 (0.00)* | *1.00 [1.00-1.50]* | *0 (0.00)* | *19(76)* | *6(24)* | *0 (0.00)* | *0 (0.00)* | *0.124* |
| *Tw7* | *1.00 [1.00-1.25* | *2(7.69)* | *18(69.23)* | *6(23.08)* | *0 (0.00)* | *0 (0.00)* | *1.00 [1.00-2.00]* | *0 (0.00)* | *18(72)* | *7(28)* | *0 (0.00)* | *0 (0.00)* | *0.418* |
| *Tw8* | *1.00 [1.00-1.00]* | *1(3.85)* | *21(80.77)* | *4(15.38)* | *0 (0.00)* | *0 (0.00)* | *1.00 [1.00-1.00]* | *0 (0.00)* | *20(80)* | *5(20)* | *0 (0.00)* | *0 (0.00)* | *0.471* |
| *Tw9* | *1.00 [1.00-1.00]* | *4(15.38)* | *19(73.08)* | *2(7.69)* | *1(3.84)* | *0 (0.00)* | *1.00 [1.00-1.00]* | *4(16)* | *19(76)* | *2(8)* | *0 (0.00)* | *0 (0.00)* | *0.755* |
| *Tw10* | *1.00 [1.00-1.00]* | *5(19.23)* | *17(65.38)* | *4(15.38)* | *0 (0.00)* | *0 (0.00)* | *1.00 [1.00-1.00]* | *5(20)* | *18(72)* | *2(8)* | *0 (0.00)* | *0 (0.00)* | *0.617* |
| *Tw11* | *1.00 [1.00-1.00]* | *2(7.69)* | *24(92.31)* | *0 (0.00)* | *0 (0.00)* | *0 (0.00)* | *1.00 [1.00-1.00]* | *2(8)* | *23(92)* | *0 (0.00)* | *0 (0.00)* | *0 (0.00)* | *0.967* |
| *Tw12* | *1.00 [1.00-1.00]* | *2(7.69)* | *24(92.31)* | *0 (0.00)* | *0 (0.00)* | *0 (0.00)* | *1.00 [1.00-1.00]* | *3(12)* | *22(88)* | *0 (0.00)* | *0(1)* | *0 (0.00)* | *0.608* |

Median [IQR] and patient numbers (%) are reported per CTCAE grade (G0-G4). P-values represent weekly comparisons between groups.

**Supplementary Table 13: Frequencies of different CTCAE severity grades of vomiting in control group versus CoQ10 group experienced during Taxol chemotherapy**

|  | ***Control group group***  ***(n= 25)*** | | | | | | ***Coenzyme Q10 group***  ***(n= 26)*** | | | | | | ***P value*** |
| --- | --- | --- | --- | --- | --- | --- | --- | --- | --- | --- | --- | --- | --- |
|  | ***Median [IQR]*** | ***No event (%)*** | ***G1 (%)*** | ***G2 (%)*** | ***G3 (%)*** | ***G4 (%)*** | ***Median [IQR]*** | ***No event (%)*** | ***G1 (%)*** | ***G2 (%)*** | ***G3 (%)*** | ***G4 (%)*** |  |
| Tw1 | *.00 [.00-.00]* | *22(84.62)* | *4(15.38)* | *0 (0.00)* | *0 (0.00)* | *0 (0.00)* | *.00 [.00-.50]* | *19(76)* | *5(20)* | *1(4)* | *0 (0.00)* | *0 (0.00)* | *0.411* |
| *Tw2* | *.00 [.00-.25]* | *20(76.92)* | *6(23.08)* | *0 (0.00)* | *0 (0.00)* | *0 (0.00)* | *.00 [.00-.00]* | *23(92)* | *2(8)* | *0 (0.00)* | *0 (0.00)* | *0 (0.00)* | *0.155* |
| *Tw3* | *.00 [.00-.00]* | *22(84.62)* | *3(11.54)* | *1(3.85)* | *0 (0.00)* | *0 (0.00)* | *.00 [.00-.50]* | *19(76)* | *5(20)* | *1(4)* | *0 (0.00)* | *0 (0.00)* | *0.460* |
| *Tw4* | *.00 [.00-.25]* | *20(76.92)* | *6(23.08)* | *0 (0.00)* | *0 (0.00)* | *0 (0.00)* | *.00 [.00-1.00]* | *17(68)* | *8(32)* | *0 (0.00)* | *0 (0.00)* | *0 (0.00)* | *0.477* |
| *Tw5* | *.00 [.00-.25]* | *20(76.92)* | *5(19.23)* | *1(3.85)* | *0 (0.00)* | *0 (0.00)* | *.00 [.00-.50]* | *19(76)* | *5(20)* | *1(4)* | *0 (0.00)* | *0 (0.00)* | *0.938* |
| *Tw6* | *.00 [.00-.00]* | *21(80.77)* | *5(19.23)* | *0 (0.00)* | *0 (0.00)* | *0 (0.00)* | *.00 [.00-1.00]* | *16(64)* | *8(32)* | *1(4)* | *0 (0.00)* | *0 (0.00)* | *0.168* |
| *Tw7* | *.00 [.00-.00]* | *22(84.62)* | *2(7.69)* | *1(3.85)* | *1(3.85)* | *0 (0.00)* | *.00 [.00-1.00]* | *15(60)* | *10(40)* | *0 (0.00)* | *0 (0.00)* | *0 (0.00)* | *0.094* |
| *Tw8* | *.00 [.00-1.00]* | *19(73.08)* | *5(19.23)* | *2(7.69)* | *0 (0.00)* | *0 (0.00)* | *.00 [.00-1.00]* | *17(68)* | *8(32)* | *0 (0.00)* | *0 (0.00)* | *0 (0.00)* | *0.839* |
| *Tw9* | *.00 [.00-.00]* | *22(84.62)* | *3(11.54)* | *0 (0.00)* | *1(3.85)* | *0 (0.00)* | *.00 [.00-.00]* | *24(96)* | *1(4)* | *0 (0.00)* | *0 (0.00)* | *0 (0.00)* | *0.198* |
| *Tw10* | *.00 [.00-.00]* | *22(84.62)* | *3(11.54)* | *1(3.85)* | *0 (0.00)* | *0 (0.00)* | *.00 [.00-.00]* | *24(96)* | *1(4)* | *0 (0.00)* | *0 (0.00)* | *0 (0.00)* | *0.198* |
| *Tw11* | *.00 [.00-.00]* | *25(96.15)* | *1(3.85)* | *0 (0.00)* | *0 (0.00)* | *0 (0.00)* | *.00 [.00-.00]* | *23(92)* | *2(8)* | *0 (0.00)* | *0 (0.00)* | *0 (0.00)* | *0.537* |
| *Tw12* | *.00 [.00-.00]* | *25(96.15)* | *1(3.85)* | *0 (0.00)* | *0 (0.00)* | *0 (0.00)* | *.00 [.00-.00]* | *24(96)* | *1(4)* | *0 (0.00)* | *0 (0.00)* | *0 (0.00)* | *0.977* |

Data show median [IQR] and number (%) per CTCAE grade (G0-G4). Weekly group comparisons are reported using p-values.

**Supplementary Table 14: Frequencies of different CTCAE severity grades of constipation in control group versus CoQ10 group experienced during Taxol chemotherapy**

|  | *Control group group*  *(n= 25)* | | | | | | *Coenzyme Q10 group*  *(n= 26)* | | | | | | *P value* |
| --- | --- | --- | --- | --- | --- | --- | --- | --- | --- | --- | --- | --- | --- |
|  | *Median [IQR]* | *No event (%)* | *G1 (%)* | *G2 (%)* | *G3 (%)* | *G4 (%)* | *Median [IQR]* | *No event (%)* | *G1 (%)* | *G2 (%)* | *G3 (%)* | *G4 (%)* |  |
| *Tw1* | *.00 [.00-.00]]* | *26*  *(100)* | *0 (0.00)* | *0 (0.00)* | *0 (0.00)* | *0 (0.00)* | *.00 [.00-.00]]* | *24(96)* | *1(4)* | *0 (0.00)* | *0 (0.00)* | *0 (0.00)* | *0.999* |
| *Tw2* | *.00 [.00-.00]* | *24(92.31)* | *2(7.69)* | *0 (0.00)* | *0 (0.00)* | *0 (0.00)* | *.00 [.00-.00]* | *23(92)* | *2(8)* | *0 (0.00)* | *0 (0.00)* | *0 (0.00)* | *0.967* |
| *Tw3* | *.00 [.00-.00]* | *24(92.31)* | *2(7.69)* | *0 (0.00)* | *0 (0.00)* | *0 (0.00)* | *.00 [.00-.00]* | *23(92)* | *2(8)* | *0 (0.00)* | *0 (0.00)* | *0 (0.00)* | *0.967* |
| *Tw4* | *.00 [.00-.00]* | *26(100)* | *0 (0.00)* | *0 (0.00)* | *0 (0.00)* | *0 (0.00)* | *.00 [.00-.00]* | *22(88)* | *3(12)* | *0 (0.00)* | *0 (0.00)* | *0 (0.00)* | *0.999* |
| *Tw5* | *.00 [.00-1.00]* | *19(73.08)* | *5(19.23)* | *2(7.69)* | *0 (0.00)* | *0 (0.00)* | *.00 [.00-.00]* | *22(88)* | *3(12)* | *0 (0.00)* | *0 (0.00)* | *0 (0.00)* | *0.166* |
| *Tw6* | *.00 [.00-.00]* | *25(96.15)* | *1(3.85)* | *0 (0.00)* | *0 (0.00)* | *0 (0.00)* | *.00 [.00-.00]* | *24(96)* | *1(4)* | *0 (0.00)* | *0 (0.00)* | *0 (0.00)* | *0.537* |
| *Tw7* | *.00 [.00-.00]* | *23(88.46)* | *3(11.54)* | *0 (0.00)* | *0 (0.00)* | *0 (0.00)* | *.00 [.00-.00]* | *24(96)* | *0 (0.00)* | *1(4)* | *0 (0.00)* | *0 (0.00)* | *0.366* |
| *Tw8* | *.00 [.00-.00]* | *23(88.46)* | *3(11.54)* | *0 (0.00)* | *0 (0.00)* | *0 (0.00)* | *.00 [.00-.00]* | *24(96)* | *0 (0.00)* | *1(4)* | *0 (0.00)* | *0 (0.00)* | *0.366* |
| *Tw9* | *.00 [.00-.00]* | *24(92.31)* | *2(7.69)* | *0 (0.00)* | *0 (0.00)* | *0 (0.00)* | *.00 [.00-.00]* | *25(100)* | *0 (0.00)* | *0 (0.00)* | *0 (0.00)* | *0 (0.00)* | *0.999* |
| *Tw10* | *.00 [.00-.00]* | *22(84.62)* | *1(3.85)* | *3(11.54)* | *0 (0.00)* | *0 (0.00)* | *.00 [.00-.00]* | *23(92)* | *0 (0.00)* | *2(8)* | *0 (0.00)* | *0 (0.00)* | *0.440* |
| *Tw11* | *.00 [.00-.00]* | *24(92.31)* | *0 (0.00)* | *2(7.69)* | *0 (0.00)* | *0 (0.00)* | *.00 [.00-.00]* | *24(96)* | *1(4)* | *0 (0.00)* | *0 (0.00)* | *0 (0.00)* | *0.552* |
| *Tw12* | *.00 [.00-.00]* | *26(100)* | *0 (0.00)* | *0 (0.00)* | *0 (0.00)* | *0 (0.00)* | *.00 [.00-.00]* | *24(96)* | *1(4)* | *0 (0.00)* | *0 (0.00)* | *0 (0.00)* | *0.999* |

Median [IQR] and patient distribution (%) for each CTCAE grade (G0-G4) are presented. P-values reflect weekly group differences.

**Supplementary Table 15: Frequencies of different CTCAE severity grades of dry mouth in control group versus CoQ10 group experienced during Taxol chemotherapy**

|  | *Control group group* | *(n= 25)* |  |  | *Coenzyme Q10 group* | *(n= 26)* |  |  | *P value* |
| --- | --- | --- | --- | --- | --- | --- | --- | --- | --- |
|  | *Median [IQR]* | *No event (%)* | *G1 (%)* | *G2 (%)* | *Median [IQR]* | *No event (%)* | *G1 (%)* | *G2 (%)* |  |
| *Tw1* | *.00 [.00-1.00]* | *14(53.85)* | *12(46.15)* | *0 (0.00)* | *.00 [.00-1.00]* | *16(64)* | *9(36)* | *0 (0.00)* | *0.459* |
| *Tw2* | *1.00 [.00-1.00]* | *7(26.92)* | *19(73.08)* | *0 (0.00)* | *1.00 [1.00-1.00]* | *5(20)* | *20(80)* | *0 (0.00)* | *0.559* |
| *Tw3* | *1.00 [.00-1.00]* | *11(42.31)* | *15(57.69)* | *0 (0.00)* | *1.00 [.00-1.00]* | *11(44)* | *14(56)* | *0 (0.00)* | *0.903* |
| *Tw4* | *1.00 [.00-1.00]* | *8(30.77)* | *18(69.23)* | *0 (0.00)* | *1.00 [.00-1.00]* | *8(32)* | *15(60)* | *2(8)* | *0.646* |
| *Tw5* | *1.00 [.75-1.00]* | *6(23.08)* | *20(76.92)* | *0 (0.00)* | *1.00 [.00-1.00]* | *8(32)* | *16(64)* | *1(4)* | *0.713* |
| *Tw6* | *1.00 [.00-1.00]* | *12(46.15)* | *14(53.85)* | *0 (0.00)* | *1.00 [.00-1.00]* | *10(40)* | *15(60)* | *0 (0.00)* | *0.657* |
| *Tw7* | *1.00 [.00-1.00]* | *12(46.15)* | *14(53.85)* | *0 (0.00)* | *1.00 [.00-1.00]* | *7(28)* | *16(64)* | *2(8)* | *0.080* |
| *Tw8* | *1.00 [1.00-1.00]* | *4(15.38)* | *22(84.62)* | *0 (0.00)* | *1.00 [1.00-1.00]* | *4(16)* | *19(76)* | *2(8)* | *0.535* |
| *Tw9* | *1.00 [.00-1.00]* | *10(38.46)* | *16(61.54)* | *0 (0.00)* | *1.00 [1.00-1.00]* | *5(20)* | *19(76)* | *1(4)* | *0.091* |
| *Tw10* | *1.00 [.00-1.00]* | *9(34.62)* | *14(53.85)* | *3(11.54)* | *1.00 [1.00-1.00]* | *5(20)* | *18(72)* | *2(8)* | *0.497* |
| *Tw11* | *1.00 [1.00-1.00]* | *5(19.23)* | *19(73.08)* | *2(7.69)* | *1.00 [.00-1.00]* | *11(44)* | *13(52)* | *1(4)* | *0.058* |
| *Tw12* | *1.00 [.00-1.00]* | *11(42.31)* | *15(57.69)* | *0 (0.00)* | *1.00 [.00-1.00]* | *13(52)* | *12(48)* | *0 (0.00)* | *0.486* |

Median [IQR] and patient numbers (%) are shown per CTCAE grade (G0-G2). P-values indicate differences between groups for each treatment week.

**Supplementary Table 16: Frequencies of different CTCAE severity grades of urinary tract pain in control group versus CoQ10 group experienced during Taxol chemotherapy**

|  | *Control group group*  *(n= 25)* | | | | | | *Coenzyme Q10 group*  *(n= 26)* | | | | | | *P value* |
| --- | --- | --- | --- | --- | --- | --- | --- | --- | --- | --- | --- | --- | --- |
|  | *Median [IQR]* | *No event (%)* | *G1 (%)* | *G2 (%)* | *G3 (%)* | *G4 (%)* | *Median [IQR]* | *No event (%)* | *G1 (%)* | *G2 (%)* | *G3 (%)* | *G4 (%)* |  |
| *Tw1* | *.00 [.00-1.00]* | *24(92.31)* | *7(26.92)* | *0 (0.00)* | *0 (0.00)* | *0 (0.00)* | *.00 [.00-1.00]* | *16(64)* | *9(36)* | *0 (0.00)* | *0 (0.00)* | *0 (0.00)* | *0.486* |
| *Tw2* | *.00 [.00-1.00]* | *19(73.08)* | *12(46.15)* | *0 (0.00)* | *0 (0.00)* | *0 (0.00)* | *1.00 [.00-.00]* | *12(48)* | *13(52)* | *0 (0.00)* | *0 (0.00)* | *0 (0.00)* | *0.676* |
| *Tw3* | *.00 [.00-1.00]* | *22(84.62)* | *9(34.62)* | *0 (0.00)* | *0 (0.00)* | *0 (0.00)* | *.00 [.00-1.00]* | *17(68)* | *8(32)* | *0 (0.00)* | *0 (0.00)* | *0 (0.00)* | *0.843* |
| *Tw4* | *.00 [.00-1.00]* | *20(76.92)* | *9(34.62)* | *0 (0.00)* | *0 (0.00)* | *0 (0.00)* | *.00 [.00-1.00]* | *13(52)* | *11(44)* | *1(4)* | *0 (0.00)* | *0 (0.00)* | *0.289* |
| *Tw5* | *.00 [.00-1.00]* | *18(69.23)* | *9(34.62)* | *1(3.85)* | *0 (0.00)* | *0 (0.00)* | *.00 [.00-1.00]* | *15(60)* | *9(36)* | *1(4)* | *0 (0.00)* | *0 (0.00)* | *0.911* |
| *Tw6* | *1.00 [.00-1.00]* | *14(53.85)* | *14(53.85)* | *0 (0.00)* | *0 (0.00)* | *0 (0.00)* | *1.00 [.00-1.00]* | *11(44)* | *13(52)* | *1(4)* | *0 (0.00)* | *0 (0.00)* | *0.760* |
| *Tw7* | *.00 [.00-1.00]* | *19(73.08)* | *8(30.77)* | *1(3.85)* | *0 (0.00)* | *0 (0.00)* | *1.00 [.00-1.00]]* | *12(48)* | *10(40)* | *3(12)* | *0 (0.00)* | *0 (0.00)* | *0.171* |
| *Tw8* | *.00 [.00-1.00]* | *20(76.92)* | *8(30.77)* | *0 (0.00)* | *0 (0.00)* | *0 (0.00)* | *.00 [.00-1.00]* | *13(52)* | *10(40)* | *2(8)* | *0 (0.00)* | *0 (0.00)* | *0.157* |
| *Tw9* | *.00 [.00-.25]* | *23(88.46)* | *5(19.23)* | *1(3.85)* | *0 (0.00)* | *0 (0.00)* | *.00 [.00-1.00]* | *15(60)* | *9(36)* | *1(4)* | *0 (0.00)* | *0 (0.00)* | *0.217* |
| *Tw10* | *.00 [.00-.25]* | *25(96.15)* | *4(15.38)* | *2(7.69)* | *0 (0.00)* | *0 (0.00)* | *.00 [.00-.50]* | *19(76)* | *6(24)* | *0 (0.00)* | *0 (0.00)* | *0 (0.00)* | *0.938* |
| *Tw11* | *.00 [.00-.00]* | *24(92.31)* | *2(7.69)* | *0 (0.00)* | *0 (0.00)* | *0 (0.00)* | *.00 [.00-.00]* | *20(80)* | *5(20)* | *0 (0.00)* | *0 (0.00)* | *0 (0.00)* | *0.217* |
| *Tw12* | *.00 [.00-.00]* | *24(92.31)* | *2(7.69)* | *0 (0.00)* | *0 (0.00)* | *0 (0.00)* | *.00 [.00-.00]* | *21(84)* | *4(16)* | *0 (0.00)* | *0 (0.00)* | *0 (0.00)* | *0.367* |

Values are median [IQR] and number (%) per CTCAE grade (G0-G4). Weekly comparisons between groups are provided with p-values.

| **Supplementary Table 17: Frequencies of CTCAE severity grades of Alopecia** | | | |
| --- | --- | --- | --- |
| **Characteristic** | **Control Group (n=26)** | **CoQ10 Group**  **(n=25)** | **P-value** |
| **At Baseline** | | | |
| Grade 1 | 10 (26.9%) | 7(36.0%) | 0.428^a^ |
| Grade 2 | 16 (73.1%) | 18 (64.0%) |  |
| **After 12th week** | | | |
| Grade 1 | 8 (11.5%%) | 11 (44.0%%) | 0.329^a^ |
| Grade 2 | 18 (88.5%%) | 14 (56.0%%) |  |
| ^a^ Chi-square test. |  |  |  |
| A P-value < 0.05 was considered statistically significant. | | | |

**Supplementary Table 18: Frequencies of different CTCAE severity grades of Anemia in control group versus CoQ10 group experienced during Taxol chemotherapy**

|  | ***Control group group***  ***(n= 26)*** | | | | | | ***Coenzyme Q10 group***  ***(n= 25)*** | | | | | | ***Odds ratio***  ***[95% C.I.]*** | ***p-value*** |
| --- | --- | --- | --- | --- | --- | --- | --- | --- | --- | --- | --- | --- | --- | --- |
|  | ***Median [IQR]*** | ***No event (%)*** | ***G1 (%)*** | ***G2 (%)*** | ***G3 (%)*** | ***G4 (%)*** | ***Median [IQR]*** | ***No event (%)*** | ***G1 (%)*** | ***G2 (%)*** | ***G3 (%)*** | ***G4 (%)*** |  |  |
| After 3rd week | *1.00 [.00-1.00]* | *10(38.46)* | *12(46.15)* | *4(15.38)* | *0 (0.00)* | *0 (0.00)* | *1.00 [.00-1.00]* | *10(40)* | *12(48)* | *3(12)* | *0 (0.00)* | *0 (0.00)* |  | *0.819* |
| After 6th week | *1.00 [.75-1.00]* | *6(23.08)* | *15(57.69)* | *5(19.23)* | *0 (0.00)* | *0 (0.00)* | *1.00 [.00-1.00]* | *9(36)* | *12(48)* | *3(12)* | *1(4)* | *0 (0.00)* |  | *0.407* |
| After 9th week | *1.00 [.75-1.00]* | *6(23.08)* | *19(73.08)* | *1(3.85)* | *0 (0.00)* | *0 (0.00)* | *1.00 [.00-.50]* | *6(24)* | *15(60)* | *4(16)* | *0 (0.00)* | *0 (0.00)* |  | *0.529* |
| After 12th week | *1.00 [1.00-1.00]* | *1(3.85)* | *20(76.92)* | *3(11.54)* | *2(7.69)* | *0 (0.00)* | *1.00 [.00-1.00]* | *7(28)* | *17(68)* | *1(4)* | *0 (0.00)* | *0 (0.00)* | *0.128 )0.025-0.649(* | *0.013* |

CTCAE grades 1-4 (G1-G4) indicate increasing severity of anemia during Taxol chemotherapy, “No event” represents patients without anemia, and median [IQR] values summarize severity; odds ratios compare control and CoQ10 groups, with p<0.05 considered significant.

**Supplementary Table 19: Frequencies of different CTCAE severity grades of neutropenia in control group versus CoQ10 group experienced during Taxol chemotherapy**

|  | ***Control group group***  ***(n= 26)*** | | | | | | ***Coenzyme Q10 group***  ***(n= 25)*** | | | | | | ***p-value*** |
| --- | --- | --- | --- | --- | --- | --- | --- | --- | --- | --- | --- | --- | --- |
|  | ***Median [IQR]*** | ***No event (%)*** | ***G1 (%)*** | ***G2 (%)*** | ***G3 (%)*** | ***G4 (%)*** | ***Median [IQR]*** | ***No event (%)*** | ***G1 (%)*** | ***G2 (%)*** | ***G3 (%)*** | ***G4 (%)*** |  |
| After 3rd week | *.00 [.00-.00]* | *21(80.77)* | *3(11.54)* | *1(3.85)* | *1(3.85)* | *0 (0.00)* | *.00 [.00-.00]* | *24(96)* | *0 (0.00)* | *1(4)* | *0 (0.00)* | *0 (0.00)* | *0.132* |
| After 6th week | *.00 [.00-.00]* | *20(76.92)* | *4(15.38)* | *1(3.85)* | *1(3.85)* | *0 (0.00)* | *.00 [.00-.00]* | *22(88)* | *1(4)* | *2(8)* | *0 (0.00)* | *0 (0.00)* | *0.336* |
| After 9th week | *.00 [.00-.00]* | *22(84.62)* | *3(11.54)* | *0 (0.00)* | *1(3.85)* | *0 (0.00)* | *.00 [.00-.00]* | *25(100)* | *0 (0.00)* | *0 (0.00)* | *0 (0.00)* | *0 (0.00)* | *0.999* |
| After 12th week | *.00 [.00-.00]* | *23(88.46)* | *3(11.54)* | *0 (0.00)* | *0 (0.00)* | *0 (0.00)* | *.00 [.00-.00]* | *23(92)* | *2(8)* | *0 (0.00)* | *0 (0.00)* | *0 (0.00)* | *0.673* |

CTCAE grades 1-4 (G1-G4) indicate increasing severity of neutropenia during Taxol chemotherapy, “No event” represents patients without neutropenia, and median [IQR] values summarize severity; p-values compare control and CoQ10 groups, with p<0.05 considered significant.

**Supplementary Table 20: Frequencies of different CTCAE severity grades of Febrile neutropenia in control group versus CoQ10 group experienced during Taxol chemotherapy**

|  | ***Control group group***  ***(n= 26)*** | | | | ***Coenzyme Q10 group***  ***(n= 25)*** | | | | ***p-value*** |
| --- | --- | --- | --- | --- | --- | --- | --- | --- | --- |
|  | ***Median [IQR]*** | ***No event (%)*** | ***G3 (%)*** | ***G4 (%)*** | ***Median [IQR]*** | ***No event (%)*** | ***G3 (%)*** | ***G4 (%)*** |  |
| After 3rd week | *.00 [.00-.00]* | *24(92.31)* | *2(7.69)* | *0 (0.00)* | *.00 [.00-.00]* | *25(100)* | *0 (0.00)* | *0 (0.00)* | *0.999* |
| After 6th week | *.00 [.00-.00]* | *25(96.15)* | *1(3.85)* | *0 (0.00)* | *.00 [.00-.00]* | *25(100)* | *0 (0.00)* | *0 (0.00)* | *0.999* |
| After 9th week | *.00 [.00-.00]* | *25(96.15)* | *1(3.85)* | *0 (0.00)* | *.00 [.00-.00]* | *25(100)* | *0 (0.00)* | *0 (0.00)* | *0.999* |
| After 12th week | *.00 [.00-.00]* | *26(100)* | *0 (0.00)* | *0 (0.00)* | *.00 [.00-.00]* | *25(100)* | *0 (0.00)* | *0 (0.00)* | *NA* |

CTCAE grades 3-4 (G3-G4) indicate severity of febrile neutropenia during Taxol chemotherapy, “No event” represents patients without febrile neutropenia, and median [IQR] values summarize severity; p-values compare control and CoQ10 groups, with p<0.05 considered significant.

**Supplementary Table 21: Frequencies of different CTCAE severity grades of thrombocytopenia in control group versus CoQ10 group experienced during Taxol chemotherapy**

|  | ***Control group group***  ***(n= 26)*** | | | | | | ***Coenzyme Q10 group***  ***(n= 25)*** | | | | | | ***p-value*** |
| --- | --- | --- | --- | --- | --- | --- | --- | --- | --- | --- | --- | --- | --- |
|  | ***Median [IQR]*** | ***No event (%)*** | ***G1 (%)*** | ***G2 (%)*** | ***G3 (%)*** | ***G4 (%)*** | ***Median [IQR]*** | ***No event (%)*** | ***G1 (%)*** | ***G2 (%)*** | ***G3 (%)*** | ***G4 (%)*** |  |
| After 3rd week | *.00 [.00-.00]* | 26(100) | 0 (0.00) | 0 (0.00) | 0 (0.00) | 0 (0.00) | *.00 [.00-.00]* | 24(96) | 1(4) | 0 (0.00) | 0 (0.00) | 0 (0.00) | *0.999* |
| After 6th week | *.00 [.00-.00]* | 25(96.15) | 1(3.85) | 0 (0.00) | 0 (0.00) | 0 (0.00) | *.00 [.00-.00]* | 25(100) | 0 (0.00) | 0 (0.00) | 0 (0.00) | 0 (0.00) | *0.999* |
| After 9th week | *.00 [.00-.00]* | 22(84.62) | 4(15.38) | 0 (0.00) | 0 (0.00) | 0 (0.00) | *.00 [.00-.00]* | 24(96) | 1(4) | 0 (0.00) | 0 (0.00) | 0 (0.00) | *0.203* |
| After 12th week | *.00 [.00-.00]* | 26(100) | 0 (0.00) | 0 (0.00) | 0 (0.00) | 0 (0.00) | *.00 [.00-.00]* | 25(100) | 0 (0.00) | 0 (0.00) | 0 (0.00) | 0 (0.00) | *NA* |

CTCAE grades 1-4 (G1-G4) indicate increasing severity of thrombocytopenia during Taxol chemotherapy, “No event” represents patients without thrombocytopenia, and median [IQR] values summarize severity; p-values compare control and CoQ10 groups, with p<0.05 considered significant.

**Supplementary Table 22. Comparison of Per-Protocol and Intention-to-Treat (ITT-LOCF) analyses of CoQ10 effects on paclitaxel-induced toxicities**

| **Toxicity** | **Week** | **Per Protocol (PP) OR (95% ci)** | **PP p-value** | **Intention to treat (ITT-LOCF) OR (95% ci)** | **ITT p-value** |
| --- | --- | --- | --- | --- | --- |
| Peripheral neuropathy | 10 | 0.162 (0.046-0.565) | 0.004 | 0.162 (0.046-0.565) | 0.004 |
|  | 11 | 0.105 (0.022-0.555) | 0.008 | 0.189 (0.059-0.604) | 0.005 |
|  | 12 | 0.107 (0.030-0.384) | 0.001 | 0.088 (0.026-0.293) | <0.001 |
| Arthralgia | 11 | 0.060 (0.012-0.298) | 0.001 | 0.189 (0.059-0.604) | 0.005 |
|  | 12 | 0.109 (0.027-0.441) | 0.002 | 0.055 (0.011-0.263) | 0.000 |
| Myalgia | 11 | 0.235 (0.070-0.783) | 0.018 | 0.240 (0.080-0.724) | 0.011 |
|  | 12 | 0.153 (0.043-0.549) | 0.004 | 0.132 (0.039-0.455) | 0.001 |
| Fatigue | 11 | 0.027 (0.005-0.143) | 0.000 | 0.081 (0.025-0.265) | 0.000 |
|  | 12 | 0.024 (0.004-0.127) | 0.000 | 0.058 (0.016-0.206) | 0.000 |
| Headache | 10 | 0.315 (0.113-0.880) | 0.028 | 0.315 (0.113-0.880) | 0.028 |
|  | 11 | 0.267 (0.100-0.714) | 0.009 | 0.267 (0.100-0.714) | 0.009 |
|  | 12 | 0.233 (0.085-0.636) | 0.004 | 0.233 (0.085-0.636) | 0.004 |
| Insomnia | 10 | 0.320 (0.120-0.853) | 0.023 | 0.320 (0.120-0.853) | 0.023 |
|  | 11 | 0.048 (0.012-0.190) | 0.000 | 0.127 (0.044-0.369) | 0.000 |
|  | 12 | 0.034 (0.008-0.150) | 0.000 | 0.096 (0.032-0.289) | 0.000 |
| Diarrhea | 11 | 0.099 (0.026-0.379) | 0.001 | 0.130 (0.036-0.461) | 0.002 |
|  | 12 | 0.121 (0.032-0.459) | 0.002 | 0.154 (0.043-0.549) | 0.004 |
| Mucositis | 11 | 0.050 (0.010-0.257) | 0.000 | 0.284 (0.099-0.812) | 0.019 |
|  | 12 | 0.090 (0.022-0.377) | 0.001 | 0.209 (0.071-0.618) | 0.005 |

Odds ratios (OR) and 95% confidence intervals (CI) are shown for each toxicity at weeks 10-12. Per-Protocol (PP) analysis includes participants who completed the study according to protocol. Intention-to-Treat (ITT-LOCF) analysis includes all randomized participants, with missing values imputed using the Last Observation Carried Forward method.

**Supplementary Table 23: Sensitivity analysis of CoQ10 effects on paclitaxel-induced toxicities-best-case and worst-case scenarios**

| **Toxicity** | **Week** | **Best-case OR (95% CI)** | **Best-case p-value** | **Worst-case OR (95% CI)** | **Worst-case p-value** |
| --- | --- | --- | --- | --- | --- |
| Peripheral neuropathy | 10 | 0.090 (0.023-0.349) | 0.001 | 1.133 (0.418-3.071) | 0.806 |
|  | 11 | 0.054 (0.011-0.263) | 0.000 | 0.925 (0.341-2.512) | 0.879 |
|  | 12 | 0.062 (0.018-0.215) | 0.000 | 0.709 (0.272-1.847) | 0.482 |
| Arthralgia | 11 | 0.061 (0.015-0.237) | 0.000 | 0.689 (0.264-1.800) | 0.447 |
|  | 12 | 0.035 (0.007-0.169) | 0.000 | 0.538 (0.204-1.416) | 0.209 |
| Myalgia | 11 | 0.122 (0.038-0.387) | 0.000 | 0.965 (0.373-2.498) | 0.942 |
|  | 12 | 0.083 (0.024-0.288) | 0.000 | 0.755 (0.292-1.954) | 0.562 |
| Fatigue | 9 | 0.116 (0.036-0.370) | 0.000 | 0.724 (0.286-1.830) | 0.495 |
|  | 10 | 0.112 (0.037-0.344) | 0.000 | 0.755 (0.292-1.953) | 0.563 |
|  | 11 | 0.017 (0.003-0.090) | 0.000 | 0.310 (0.117-0.826) | 0.019 |
|  | 12 | 0.015 (0.003-0.081) | 0.000 | 0.292 (0.109-0.781) | 0.014 |
| Headache | 10 | 0.068 (0.020-0.230) | 0.000 | 0.689 (0.265-1.788) | 0.444 |
|  | 11 | 0.064 (0.019-0.211) | 0.000 | 0.459 (0.180-1.169) | 0.103 |
|  | 12 | 0.053 (0.015-0.188) | 0.000 | 0.441 (0.172-1.133) | 0.089 |
| Insomnia | 9 | 0.099 (0.033-0.299) | 0.000 | 0.724 (0.286-1.830) | 0.495 |
|  | 10 | 0.086 (0.028-0.263) | 0.000 | 0.621 (0.246-1.569) | 0.313 |
|  | 11 | 0.031 (0.008-0.120) | 0.000 | 0.328 (0.125-0.863) | 0.024 |
|  | 12 | 0.022 (0.005-0.096) | 0.000 | 0.289 (0.109-0.765) | 0.012 |
| Diarrhea | 11 | 0.059 (0.016-0.219) | 0.000 | 0.546 (0.202-1.473) | 0.232 |
|  | 12 | 0.070 (0.019-0.254) | 0.000 | 0.652 (0.240-1.767) | 0.400 |
| Mucositis | 11 | 0.032 (0.006-0.164) | 0.000 | 0.359 (0.122-1.055) | 0.063 |
|  | 12 | 0.057 (0.014-0.232) | 0.000 | 0.487 (0.170-1.392) | 0.179 |

Odds ratios (OR) and 95% confidence intervals (CI) are shown for each toxicity at the specified weeks. The best-case scenario assumes the most favorable outcomes for missing or excluded participants, while the worst-case scenario assumes the least favorable outcomes.

| **Adverse events assessment form according to the National Cancer Institute Common Terminology Criteria for Adverse Events (NCI-CTCAE) version 5.0** (1) | | | | | | |
| --- | --- | --- | --- | --- | --- | --- |
| **Patient name:** | |  | | | | |
| **Patient ID:** | |  | | | | |
| **Clinician:** | |  | | | | |
| **Date:** | |  | | | | |
| **Cycle number:** | | **Cycle 1** | **Cycle 2** | **Cycle 3** | **Cycle 4** | |
|  |  | **Tw 1** | **Tw 4** | **Tw 7** | **Tw 10** | |
|  |  | **Tw 2** | **Tw 5** | **Tw 8** | **Tw 11** | |
|  |  | **Tw 3** | **Tw 6** | **Tw 9** | **Tw 12** | |
| **CTCAE v5.0 SOC** | **CTCAE v5.0 Term** | **Grade 1** | **Grade 2** | **Grade 3** | **Grade 4** | **Grade 5** |
| **General disorders and administration site conditions** | **Fatigue** | Fatigue relieved by rest | Fatigue not relieved by rest; limiting instrumental ADL | Fatigue not relieved by rest, limiting self-care ADL | - | - |
|  | A disorder characterized by a state of generalized weakness with a pronounced inability to summon sufficient energy to accomplish daily activities. | | | | | |
| **Nervous system disorders** | **Headache** | Mild pain | Moderate pain; limiting instrumental ADL | Severe pain; limiting self-care ADL | - | - |
|  | A disorder characterized by a sensation of marked discomfort in various parts of the head, not confined to the area of distribution of any nerve. | | | | | |
|  | **Insomnia** | Mild difficulty falling asleep, staying asleep or waking up early | Moderate difficulty falling asleep, staying asleep or waking up early | Severe difficulty in falling asleep, staying asleep or waking up early | - | - |
|  | A disorder characterized by difficulty in falling asleep and/or remaining asleep. | | | | | |
|  | **Peripheral sensory neuropathy** | Asymptomatic | Moderate symptoms; limiting instrumental ADL | Severe symptoms; limiting self-care ADL | Life-threatening consequences: urgent intervention indicated |  |
|  | A disorder characterized by damage or dysfunction of the peripheral sensory nerves. | | | | | |
| **Musculoskeletal and connective tissue disorders** | **Arthralgia** | Mild pain | Moderate pain; limiting instrumental ADL | Severe pain; limiting self-care ADL | - | - |
|  | A disorder characterized by a sensation of marked discomfort in a joint. | | | | | |
|  | **Myalgia** | Mild pain | Moderate pain; limiting instrumental ADL | Severe pain; limiting self-care ADL | - | - |
|  | A disorder characterized by marked discomfort sensation originating from a muscle or group of muscles. | | | | | |
| **Gastrointestinal disorders** | **Nausea** | Loss of appetite without alteration in eating habits | Oral intake decreased without significant weight loss, dehydration or malnutrition | Inadequate oral caloric or fluid intake; tube feeding, TPN, or hospitalization indicated | - | - |
|  | A disorder characterized by a queasy sensation and/or the urge to vomit. | | | | | |
|  | **Vomiting** | Intervention not indicated | Outpatient IV hydration; medical intervention indicated | Tube feeding, TPN, or hospitalization indicated | Life-threatening consequences | Death |
|  | A disorder characterized by the reflexive act of ejecting the contents of the stomach through the mouth. | | | | | |
|  | **Diarrhea** | Increase of <4 stools per day over baseline; mild increase in ostomy output compared to baseline | Increase of 4 - 6 stools per day over baseline; moderate increase in ostomy output compared to baseline | Increase of >=7 stools per day over baseline; incontinence; hospitalization indicated; severe increase in ostomy output compared to baseline; limiting self-care ADL | Life-threatening consequences: urgent intervention indicated | Death |
|  | A disorder characterized by frequent and watery bowel movements. | | | | | |
|  | **Constipation** | Occasional or intermittent symptoms; occasional use of stool softeners, laxatives, dietary modification, or enema | Persistent symptoms with regular use of laxatives or enemas; limiting instrumental ADL | Obstipation with manual evacuation indicated, limiting self-care ADL | Life-threatening consequences: urgent intervention indicated | Death |
|  | A disorder characterized by irregular and infrequent or difficult evacuation of the bowels. | | | | | |
|  | **oral Mucositis** | Asymptomatic or mild symptoms; intervention not indicated | Moderate pain; not interfering with oral intake; modified diet indicated | Severe pain; interfering with oral intake | Life-threatening consequences: urgent intervention indicated | Death |
|  | A disorder characterized by inflammation of the oral mucosal. | | | | | |
|  | **Dry mouth** | Symptomatic (e.g., dry or thick saliva) without significant dietary alteration; unstimulated saliva flow >0.2 ml/min | Moderate symptoms; oral intake alterations (e.g., copious water, other lubricants, diet limited to purees and/or soft, moist foods); unstimulated saliva 0.1 to 0.2 ml/min | Inability to adequately aliment orally; tube feeding or TPN indicated; unstimulated saliva <0.1 ml/min | - | - |
|  | A disorder characterized by reduced salivary flow in the oral cavity. | | | | | |
| **Renal and urinary disorders** | **Urinary tract pain** | Mild pain | Moderate pain; limiting instrumental ADL | Severe pain; limiting self-care ADL | - | - |
|  | A disorder characterized by a sensation of marked discomfort in the urinary tract. | | | | | |
| **Blood and lymphatic system disorders** | **Anemia** | Hemoglobin (Hgb) <LLN - 10.0 g/dL; <LLN - 6.2 mmol/L; <LLN - 100 g/L | Hgb <10.0 - 8.0 g/dL; <6.2 - 4.9 mmol/L; <100 - 80g/L | Hgb <8.0 g/dL; <4.9 mmol/L; <80 g/L; transfusion indicated | Life-threatening consequences: urgent intervention indicated | Death |
|  | A disorder characterized by an reduction in the amount of hemoglobin in 100 ml of blood. Signs and symptoms of anemia may include pallor of the skin and mucous membranes, shortness of breath, palpitations of the heart, soft systolic murmurs, lethargy, and fatigability. | | | | | |
|  | **Neutrophil count decreased** | <LLN - 1500/mm3; <LLN - 1.5 x 10e9 /L | <1500 - 1000/mm3; <1.5 - 1.0 x 10e9 /L | <1000 - 500/mm3; <1.0 - 0.5 x 10e9 /L | <500/mm3; <0.5 x 10e9 /L | - |
|  | A finding based on laboratory test results indicate a decrease in number of neutrophils in a blood specimen. | | | | | |
|  | **Platelet count decreased** | <LLN - 75,000/mm3; <LLN - 75.0 x 10e9 /L | <75,000 - 50,000/mm3; <75.0 - 50.0 x 10e9 /L | <50,000 - 25,000/mm3; <50.0 - 25.0 x 10e9 /L | <25,000/mm3; <25.0 x 10e9 /L | - |
|  | A finding based on laboratory test results that indicate a decrease in number of platelets in a blood specimen. | | | | | |
|  | **Febrile neutropenia** | - | - | ANC <1000/mm3 with a single temperature of >38.3 degrees C (101 degrees F) or a sustained temperature of >=38 degrees C (100.4 degrees F) for more than one hour | Life-threatening consequences: urgent intervention indicated | Death |
|  | A disorder characterized by an ANC <1000/mm3 and a single temperature of >38.3 degrees C (101 degrees F) or a sustained temperature of >=38 degrees C (100.4 degrees F) for more than one hour. | | | | | |
| **Skin and subcutaneous tissue disorders** | **Alopecia** | Hair loss of <50% of normal for that individual that is not obvious from a distance but only on close inspection; a different hair style may be required to cover the hair loss but it does not require a wig or hair piece to camouflage | Hair loss of >=50% normal for that individual that is readily apparent to others; a wig or hair piece is necessary if the patient desires to completely camouflage the hair loss; associated with psychosocial impact |  |  |  |
|  | A disorder is characterized by a decrease in density of hair compared to normal for a given individual at a given age and body location. | | | | | |
|  | **Nail changes** | Present | - | - | - | - |
|  | A disorder characterized by a change in the nails. | | | | | |

1. CTEP Trial Development and Conduct - NCI [Internet]. 2025 [cited 2025 Nov 9]. Available from: https://dctd.cancer.gov/research/ctep-trials/trial-development
